# THOC7-AS1/OCT1/FSTL1 axis promotes EMT and serves as a therapeutic target in cutaneous squamous cell carcinoma

**DOI:** 10.1186/s12967-024-05116-8

**Published:** 2024-04-11

**Authors:** Site Yu, Xu Cui, Situo Zhou, Yun Li, Wenjie Feng, Xiangjun Zhang, Yuhui Zhong, Pihong Zhang

**Affiliations:** Department of Burns and Plastic Surgery, Xiangya Hospital, Central South University, 87 Xiangya Road, Changsha, Hunan Province 410008 P.R. China

**Keywords:** THOC7-AS1, OCT1, FSTL1, cSCC, EMT, Targeted therapy

## Abstract

**Background:**

THOC7-AS1 and FSTL1 expression are frequently upregulated in cutaneous squamous cell carcinoma (cSCC). However, their molecular biological mechanisms remain elusive and their potential as therapeutic targets needs urgent exploration.

**Methods:**

Human tissue samples were used to evaluate clinical parameters. In vitro and in vivo experiments assessed biological functions. Quantitative PCR, western blot, immunohistochemistry, immunocytochemistry, immunoprecipitation, RNA fluorescence in situ hybridization, RNA pull-down, RNA immunoprecipitation, silver staining, chromatin immunoprecipitation, dual luciferase reporter assays etc. were utilized to explore the molecular biological mechanisms.

**Results:**

We found FSTL1 is an oncogene in cSCC, with high expression in tumor tissues and cells. Its elevated expression closely associates with tumor size and local tissue infiltration. In vitro and in vivo, high FSTL1 expression promotes cSCC proliferation, migration and invasion, facilitating malignant behaviors. Mechanistically, FSTL1 interacts with ZEB1 to promote epithelial-to-mesenchymal transition (EMT) in cSCC cells. Exploring upstream regulation, we found THOC7-AS1 can interact with OCT1, which binds the FSTL1 promoter region and promotes FSTL1 expression, facilitating cSCC progression. Finally, treating tumors with THOC7-AS1 antisense oligonucleotides inhibited cSCC proliferative and migratory abilities, delaying tumor progression.

**Conclusions:**

The THOC7-AS1/OCT1/FSTL1 axis regulates EMT and promotes tumor progression in cSCC. This study provides clues and ideas for cSCC targeted therapy.

**Supplementary Information:**

The online version contains supplementary material available at 10.1186/s12967-024-05116-8.

## Background

Cutaneous squamous cell carcinoma (cSCC) is the second most common skin tumor after basal cell carcinoma (excluding melanoma), accounting for 20-50% of skin tumors [1]. According to the statistics, approximately 200,000-400,000 new cases of cSCC are diagnosed annually in the United States, resulting in around 3,000 deaths [2]. With advancements in diagnosis and treatment, the incidence of cSCC is rising worldwide [3]. Advanced poorly differentiated cSCC is highly invasive, progresses rapidly, and often causes invasion into adjacent organs and distant metastasis. Treatment options are frequently limited, seriously impacting patient survival and quality of life [4]. Currently, cSCC treatment still follows the latest Guidelines for Squamous Cell Carcinoma of the Skin from the American Academy of Dermatology (AAD). Recommended modalities include surgery, photodynamic therapy (PDT), topical drug therapy, radiation, cryotherapy, and laser therapy [5]. However, many patients have inoperable cSCC, distant metastases, or radioresistance, rendering these approaches ineffective [6]. Exploring the molecular mechanisms underlying cSCC and identifying viable diagnostic and therapeutic targets is thus of great clinical and scientific value.

The FSTL1 gene encodes a secreted 35 kDa protein, structurally akin to follistatin, primarily expressed in the endoplasmic reticulum and extracellular space [7]. Follistatin-like 1 (FSTL1) exhibits altered expression in various health conditions, including cardiovascular diseases, arthritis, and cancer [8]. Previous investigations have unveiled FSTL1’s involvement in diverse signaling pathways and biological processes such as angiogenesis, immune regulation, cell proliferation, migration, and inflammation [8]. The dynamic expression of FSTL1 in these contexts underscores its multifaceted roles, positioning it as a pivotal contributor to a range of physiological and pathological mechanisms. In the realm of cancer, FSTL1 has been implicated in gastric cancer progression by promoting cell proliferation and metastasis through the activation of the AKT signaling pathway, which is indicative of a poor prognosis [9]. Additionally, activated tumor-associated fibroblasts have been identified as secretors of FSTL1, influencing hepatocellular carcinoma cells to augment metastasis and stemness, thereby contributing to disease progression [10]. Despite these significant findings in other cancer types, the exploration of FSTL1 in cSCC remains largely uncharted. Given the considerable impact observed in other malignancies, a thorough investigation of FSTL1’s role in cSCC is deemed clinically imperative. This study aims to address this knowledge gap and provide valuable insights into the potential involvement of FSTL1 in cSCC pathogenesis.

OCT1, also known as POU2F1, was one of the first transcription factors identified in the POU family, containing the 160-amino acid POU domain required for DNA binding to the octameric sequence ATGCAAAT [11]. OCT1 was initially found to regulate proliferation and immunomodulation, but recent evidence shows additional roles in stem cell viability, oxidative/cytotoxic resistance and metabolic control [12–14]. OCT1 exhibits pro-carcinogenic effects in various contexts. Numerous reports demonstrate the prognostic and therapeutic value of OCT1 transcriptional targets across diverse malignancies including breast cancer, lung cancer, colorectal cancers and so on [15, 16]. For instance, in glioblastoma multiforme, hypoxic conditions induce OCT1 interaction with the lncRNA MIR210HG, regulating the oncogenes IGFBP2 and FGFR1, resulting in poor prognosis [17]. Despite the wealth of evidence in various cancers, the exploration of OCT1’s role in cSCC remains limited. This gap in knowledge prompts the need for an in-depth investigation to unravel the potential implications of OCT1 in cSCC pathogenesis, prognosis, and therapeutic strategies.

LncRNAs are transcripts over 200 nucleotides that do not encode proteins. While some may represent transcriptional “noise,” many possess unique functionalities influencing virtually all cellular activities [18, 19]. With tens of thousands of lncRNAs discovered, determining their subcellular localization is critical for elucidating their biological properties [20, 21]. LncRNAs are major regulators of chromatin dynamics and gene expression, interacting with diverse signaling pathways. Their aberrant expression, mutations, and SNPs have been linked to tumorigenesis and metastasis [22]. For instance, lncROPM is upregulated in breast tumors, correlating with advanced staging and poor prognosis by maintaining cancer stemness through PLA2G16-mediated activation of PI3K/AKT, Wnt/β-catenin, and Hippo/YAP signaling [23]. THOC7-AS1 is a lncRNA with unknown expression, localization, and function in tumors. The exploration of its biological significance in cSCC is thus a warranted avenue, providing an opportunity to unravel its potential roles in the intricate molecular landscape of this skin cancer.

During primary tumorigenesis, progression, and metastasis, cancer cells undergo transitional states characterized by plasticity, including epithelial-mesenchymal transition (EMT) and mesenchymal-epithelial transition (MET) [24]. EMT causes cells to lose epithelial traits and integrity while acquiring mesenchymal features and motility [25]. Aberrant EMT reactivation enables key morphological and motility changes that drive invasion in cancer [26]. Moreover, EMT orchestrates complementary malignancy characteristics like stemness, tumorigenicity, drug resistance, and adaptation to microenvironmental changes [27]. For example, in HaCaT cells, MALAT1 facilitates TGF-β1-mediated EMT by upregulating the EMT transcription factor ZEB1 [28]. MAGE-C3 promotes metastasis in esophageal squamous cell carcinoma by inducing EMT and immunosuppression [29]. It’s noteworthy that the epithelial-mesenchymal transition of tumor cells may also occur during the progression of cSCC. This process underscores the importance of understanding the molecular mechanisms driving transitions between epithelial and mesenchymal states, as it has significant implications for the invasive and metastatic potential of cSCC.

In our investigation, we unveiled the oncogenic role of FSTL1 in cutaneous squamous cell carcinoma. Notably, FSTL1 expression was found to be elevated in both cSCC cells and tissues, exhibiting a strong correlation with poor prognosis. Functionally, the reduction of FSTL1 expression demonstrated inhibitory effects on proliferation, migration, and invasion in both in vitro and in vivo settings. Mechanistically, our findings revealed that FSTL1 promotes epithelial-mesenchymal transition (EMT) in cSCC by engaging in an interaction with the EMT transcription factor ZEB1. Furthermore, our study identified an upregulation of THOC7-AS1 expression in cSCC. THOC7-AS1 was observed to interact with OCT1, a transcription factor that binds to the FSTL1 promoter, thereby promoting its expression. Intriguingly, antisense oligonucleotides targeting THOC7-AS1 demonstrated substantial anti-tumor effects in both in vitro and in vivo experimental setups. Collectively, our data shed light on the regulatory axis involving THOC7/OCT1/FSTL1, elucidating its pivotal role in governing EMT and driving the progression of cSCC. These findings unveil new perspectives on potential therapeutic targets for cSCC.

Late-stage cutaneous squamous cell carcinoma is characterized by strong invasiveness, rapid progression, and a high risk of recurrence and metastasis. Patients at this stage often lose the opportunity for surgical intervention. Currently, systemic treatment options are relatively limited, primarily consisting of cytotoxic chemotherapy and EGFR inhibitor therapy [30]. Clinical and preclinical trials suggest that PD-1 immune checkpoint inhibitors may have a certain role in the treatment of advanced cSCC, although novel therapeutic strategies are scarce [30]. Targeted treatment options for cSCC are limited, necessitating the urgent exploration of new therapeutic targets. Therefore, this study aims to explore potential treatment targets for cSCC from a clinical perspective. FSTL1, identified as a key signaling molecule in our cSCC research, has been previously recognized for its crucial role in the progression of gastric and hepatic cancers [8, 9]. However, its regulatory mechanisms in cutaneous squamous cell carcinoma remain unclear. Hence, our experiments will further investigate and elucidate the expression and regulatory mechanisms of FSTL1, along with its pro-carcinogenic effects on cSCC. Currently, certain pharmaceutical companies are exploring the role of the FSTL1-targeting drug PFI-103 in preclinical trials for osteosarcoma and solid tumors [31]. Therefore, our experimental research is expected to further substantiate its clinical therapeutic value in cancer.

## Materials and methods

### Patients and tissue samples

Tumor specimens from 66 patients with cSCC surgically resected at Xiangya Hospital of Central South University from January 1, 2017 to August 31, 2023 were selected for this study. Immediately after excision, all specimens were placed in tissue-protecting solution and quickly transferred to liquid nitrogen for cryopreservation. Tissue samples were used to prepare immunohistochemical sections. Neoplastic and paracancerous tissues from 5 cSCC patients were also used to extract mRNA and protein. Patient clinicopathologic features were obtained from medical records. Tumor pathological diagnosis was made according to the American Joint Committee on Cancer (AJCC) TNM Classification for Cutaneous Carcinoma of the Head and Neck (8th ed., 2017). The study was approved by the Ethics Committee of Xiangya Hospital, and written informed consent was obtained from all participants.

### Public databases

cSCC datasets GSE32628 and GSE66359 were selected from the GEO database for gene selection. The TCGA and GTEx databases were used to analyze survival curves. GSE63329 was used to analyze key signaling pathways.

### Cell lines and culture

Human cSCC cell lines (A431, Colo16, SCL1) and normal human keratinocyte HaCat cells were purchased from Abiowell or BIOESN Biotech with STR certification reports. Cells were cultured in high-glucose DMEM (Gibco) supplemented with 10% fetal bovine serum and 1% penicillin/streptomycin at 37 °C with 5% CO_2_.

### Immunohistochemistry (IHC)

Immunohistochemistry was employed to evaluate the expression levels of specific markers in human cSCC tissue samples (FSTL1, ZEB1) as well as in nude mouse subcutaneous tumor tissue samples (FSTL1, Ki67, ZEB1, E-cadherin, N-cadherin, Vimentin). Sections were deparaffinized in an oven at 65 °C for 2 h. They were subsequently hydrated through a series of solutions: xylene 1, xylene 2, anhydrous ethanol, 95% ethanol, 80% ethanol, and 60% ethanol. Antigen retrieval was performed by heating sections in boiling sodium citrate solution for 10 min. After cooling to room temperature, endogenous peroxidase activity was blocked using 3% hydrogen peroxide. Sections were then incubated in 10% BSA (BioFroxx; neoFroxx GmbH) for 30 min to prevent nonspecific binding. Primary antibodies were applied and left to incubate overnight at 4 °C. The secondary antibody was applied and incubated for 1 h. Subsequently, sections were stained using DAB (3,3’-Diaminobenzidine) (Servicebio, Wuhan, China). Hematoxylin staining (Servicebio, Wuhan, China) was performed for 5 min followed by differentiation in 1% hydrochloric acid alcohol for 3 s. Sections were rinsed under running water for 5 min and dehydrated through a series of ethanol concentrations: 60%, 80%, 95%, anhydrous ethanol, xylene 2, and xylene 1. Finally, the sections were sealed with neutral resin before being photographed under an optical microscope (Nikon N2-DMi8). Staining assessment was independently conducted and graded by two investigators. Staining intensity was categorized into 0 (negative), 1 (weak), 2 (moderate), and 3 (strong). The extent of staining was stratified as 0 (0%), 1 (1–25%), 2 (26–50%), 3 (51–75%), and 4 (76–100%), representing the percentage of positively stained area within the entire tumor invasion region. The final score for FSTL1 expression was derived by combining the staining intensity with the extent of staining, resulting in a scale from 0 to 7. Samples were then categorized into two groups: Low FSTL1 expression (0–3 points) and high FSTL1 expression (4–7 points). Sections were further classified into high and low expression groups based on these criteria. Detailed information about the antibodies utilized can be found in Table S1.

### Immunocytochemistry (ICC)

Cells were washed three times with PBS and subsequently fixed with 4% paraformaldehyde (Servicebio, Wuhan, China) for 15 min. Following fixation, cells were permeabilized with 0.1% Triton X-100 (Servicebio, Wuhan, China) for 5 min. Blocking was performed using 5% BSA (BioFroxx; neoFroxx GmbH) for 30 min. Primary antibodies were incubated overnight at 4 °C. The fluorescent secondary antibody was then applied and incubated for 1 h. Subsequently, cells were stained with DAPI for 15 min, mounted with a neutral resin, and photographed using an inverted fluorescence microscope (Nikon ECLIPSE Ti2). To achieve co-localization of primary antibodies from the same species in immunofluorescence, we employed the Tyramide Signal Amplification (TSA) technique. Experimental procedures were carried out in accordance with the guidelines provided by the double-label multiplex immunofluorescence kit (Abiowell Biotechnology Co.Ltd) for simultaneous detection of multiple targets in the same cellular context. Detailed information about the antibodies utilized can be found in Table S1.

### Western blot

Cells and tissues were lysed in RIPA buffer (Servicebio, Wuhan, China) with protease inhibitors (Servicebio, Wuhan, China). Total proteins were extracted and separated by 10% SDS-PAGE, then transferred to PVDF membranes. Membranes were blocked with 5% skim milk (BD, USA) for 1 h at room temperature before incubating with primary and secondary antibodies. Protein bands were visualized using ECL reagent (Servicebio, Wuhan, China). Antibody information is listed in Supplementary Table S1.

### Total RNA extraction and quantitative real-time PCR

Total RNA from cells or tissues was extracted using Trizol (TransGen Biotech, China) and reverse transcribed into cDNA with the Evo M-MLV RT Kit (Accurate Biology, China). qPCR was performed on the Vii7 system (Applied Biosystems). Relative RNA expression was calculated by the -ΔΔCt method. Primer sequences are listed in Supplementary Table S2.

### Plasmid construction, lentivirus generation, and cell infection

Empty vector served as a control. Target genes were knocked down or overexpressed in cSCC cell lines using sequences listed in Supplementary Table S3. siRNA and shRNA were designed with Beijing Tsingke Biotech Co., Ltd. Overexpression plasmids were designed with WZ Biosciences Inc. Lentiviruses were generated by transfecting 293T cells with the pLKO.1, psPAX2 and pMD2.G systems. Stable knockdown or overexpression was selected with puromycin.

### Cell counting kit 8 assay (CCK8 assay)

cSCC cells were seeded in 96-well plates (2,000 cells/well). After transfection, 10 µL CCK-8 reagent (Beyotime, Shanghai) was added to each well and incubated for 2 h in the dark. Absorbance at 450 nm was measured with a SpectrumMax Plus microplate reader (Molecular Devices).

### Colony formation assay

cSCC cells were seeded in 6-well plates at 500 cells/well for 2 weeks. Cells were fixed with 4% paraformaldehyde, stained with crystal violet, and colonies were counted under a microscope.

### Scratch assay

Confluent cSCC cell monolayers in 12-well plates were scratched vertically with a sterile pipette tip. Images were captured at 0 h and 24 h post-scratch under a Nikon inverted microscope.

### Transwell assay

For the investigation of cellular invasion, the upper chambers were pre-coated with matrix gel (354,248; Corning, Inc.) following the provided instructions before cell seeding. Transwell chambers (pore size, 8 μm; Corning, Inc.) were set up with 500µL of serum-free DMEM in the upper chamber and 500µL of 20% FBS DMEM in the lower chamber. cSCC cells were seeded in the upper chamber. After 24 h, the cells were fixed using 4% paraformaldehyde, stained with crystal violet, and imaged using a Nikon microscope. Cell counting was performed.

### Flow cytometry-based cell apoptosis detection assays

Treated cells were enzymatically dissociated using EDTA-free trypsin, and subsequently collected by centrifugation. The collected cells were then resuspended in binding buffer, following the instructions provided with the Annexin V-FITC/PI kit (Y6002M, UElandy Inc). A 5 µL volume of YF®488-Annexin V was added to the buffer and gently mixed. Subsequently, another 5 µL of PI staining solution was added to the buffer and mixed gently. The mixture was incubated for 10 min at room temperature in the absence of light. Apoptosis was promptly detected using a flow cytometer with an excitation wavelength of 488 nm.

### Cell cycle analyses via flow cytometry

The treated cells underwent trypsin digestion, and the resulting supernatant was removed post-centrifugation. Following two washes by centrifugation using 1x PBS, the cells were subjected to overnight fixation with 70% cold ethanol. After removing the fixative with 1x PBS, the cells were resuspended in 250 µL of 1x PBS. A 2 µL volume of RNase A (AWR0154, Abiowell) at a concentration of 1 mg/mL was added, followed by incubation in a water bath at 37 °C for 40 min. Subsequently, 50 µL of PI staining solution (G1021, Servicebio) was added, and the cells were incubated for 20 min, shielded from light. Flow cytometry was then performed.

### Subcutaneous tumor model

Tumor cells (100 µl 10^7 cells/ml) were injected subcutaneously into the left axilla of nude mice (4 weeks old) to form subcutaneous tumors. Mice were weighed every 3 days. Tumor weight and volume were measured after mice were sacrificed. Tumor volume was calculated as (length*width^2)/2. Tumors were paraffin-embedded for H&E and immunohistochemical staining. The study was approved by the Institutional Animal Care and Use Committee of Central South University.

### Co-immunoprecipitation assay

A431 cell total protein was extracted with ice-cold RIPA buffer with a protease inhibitor cocktail (Servicebio, Wuhan, China). Cell lysates were collected and immunoprecipitated with 2 µg of antibody or IgG control. Antibody/IgG immunoprecipitates were gently rotated at 4 °C overnight. Protein A/G Agarose (Selleck, Houston, USA) was used to pull down antibodies. After three PBS washes, immunoprecipitated proteins were added to 5X sample buffer and boiled at 95 °C for 5 min.

### Fluorescence in situ hybridization (FISH)

THOC7-AS1 probes were designed and synthesized by Servicebio Technology Co., Ltd., who also provided the FISH kit. Briefly, tumor cells on coverslips were fixed in 4% paraformaldehyde and hybridized with probes overnight. Nuclei were stained with 1 µg/mL DAPI. Images were acquired on a Nikon fluorescence microscope.

### RNA pull-down assay

Biotinylated THOC7-AS1 sense/antisense probes were designed and synthesized by WZ Biosciences Inc. Probes were incubated with cell lysates and magnetic beads overnight at 4 °C. After purification, enriched proteins underwent silver staining and Western blot analysis.

### RNA binding protein immunoprecipitation (RIP)

The PureBinding® RNA Immunoprecipitation Kit (Geneseed, Guangzhou, China) was used for RIP per manufacturer’s protocol. Cell lysates were incubated with IgG control or anti-OCT1 antibody conjugated to magnetic beads. After purification, immunoprecipitated RNAs were isolated and analyzed by qPCR.

### Chromatin immunoprecipitation (ChIP)

ChIP was performed using the Chromatin Immunoprecipitation Kit (Beyotime, Shanghai, China) per manufacturer’s instructions. Cells were fixed with 1% formaldehyde and crosslinked chromatin was sonicated to 200–1000 bp fragments, which were immunoprecipitated. Protein/DNA complexes were eluted and analyzed by real-time quantitative PCR. ChIP primer sequences are shown in Supplementary Table S4.

### Dual-luciferase reporter assay

FSTL1 promoter sequences were designed and synthesized by WZ Biosciences Inc. The FSTL1 promoter was cloned into the pGL3-promoter vector. After seeding cSCC cells in 6-well plates for 48 h, the luciferase reporter plasmid and 10 ng pRL-TK Renilla plasmid were co-transfected using Lipofectamine 8000 (Beyotime, Shanghai, China). Luciferase and Renilla signals were detected 48 h post-transfection using the Dual Luciferase Reporter Kit (TransGen Biotech, China).

### Statistical analysis

Data were analyzed using GraphPad Prism 9.0. Student’s t-test compared differences between two groups. One-way ANOVA was used to analyze differences between three groups. Correlations between continuous variables were analyzed by Spearman’s correlation. Categorical data were assessed by chi-square test or Fisher’s exact test. Experiments were conducted in triplicate. *p* < 0.05 was considered statistically significant.

## Results

### FSTL1 expression is upregulated in cSCC

**Fig. 1:**
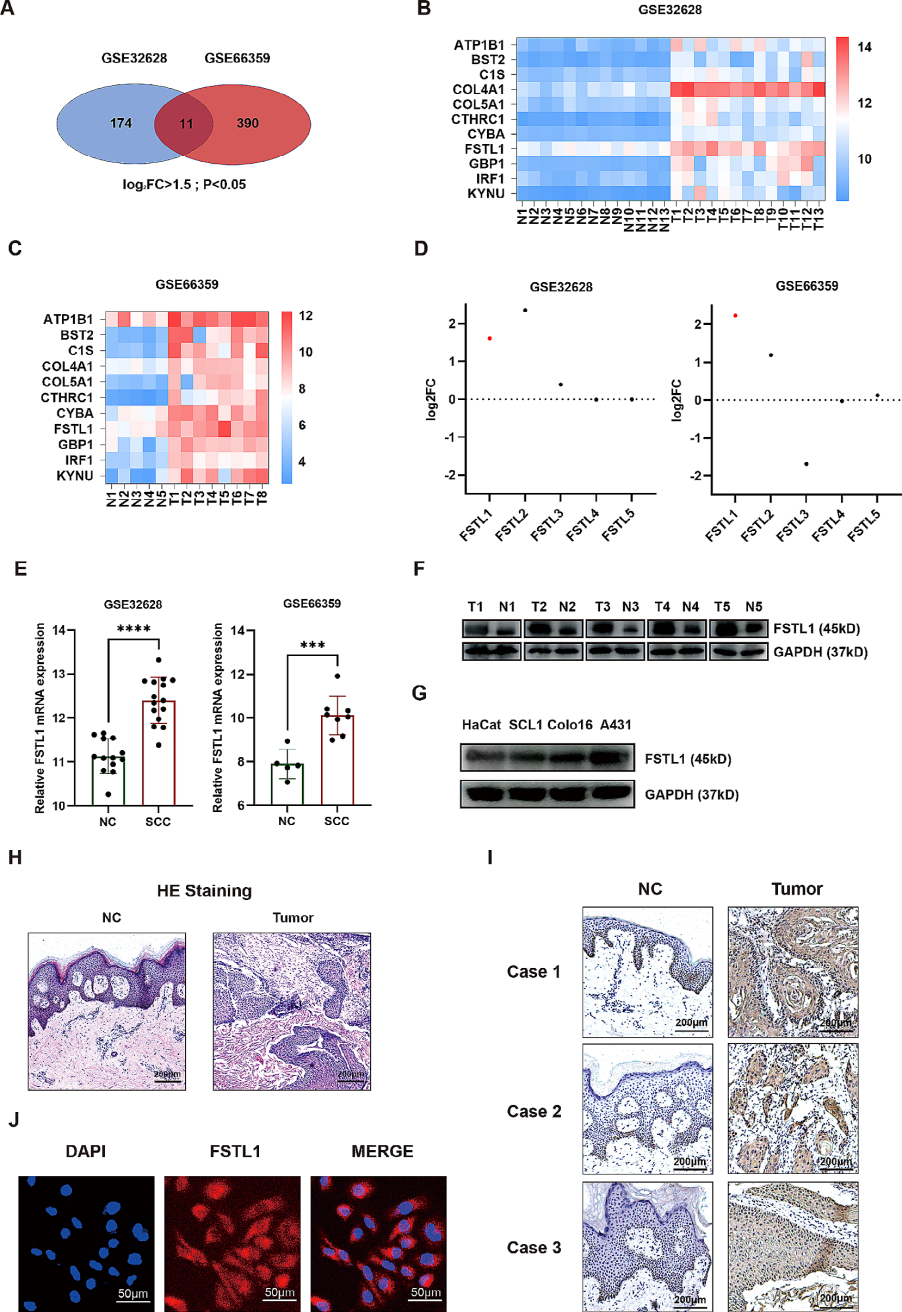
FSTL1 expression is upregulated in cSCC. (**A**) Venn diagram of differentially expressed genes in GSE32628 and GSE66359. (**B**-**C**) Heatmaps of differentially expressed gene expression in GSE32628 and GSE66359. (**D**) FSTL1-FSTL5 fold changes in the datasets. (**E**) FSTL1 mRNA expression in the datasets. (**F**) FSTL1 western blot of tissues, NC - normal control, T - tumor. (**G**) FSTL1 western blot of cell lines. (H) H&E staining of tissues, scale bars 200 μm. (**I**) FSTL1 immunohistochemistry of tissues, scale bars 200 μm. (**J**) FSTL1 immunocytochemistry in A431 cells, scale bars 50 μm. Data were presented as the mean ± SD. (ns, non-significant; *p < 0.05; **p < 0.01; ***p < 0.001; ****p < 0.0001)

In the GEO database, two cSCC datasets (GSE32628 and GSE66359) were analyzed, and through a stringent criterion of log2FC > 1.5 & *p* < 0.05, we identified 11 highly expressed genes among differentially expressed genes (Fig. 1A-C). Following a thorough review of current literature and consideration of the obtained results, FSTL1 emerged as a promising candidate for further investigation. Notably, FSTL1 expression in cutaneous squamous carcinoma was higher compared to most other family members based on the database analysis (Fig. 1D). Further validation demonstrated upregulation of FSTL1 mRNA in tumors when compared to adjacent normal tissues (Fig. 1E). Subsequently, Western blot analysis of our own samples confirmed elevated FSTL1 protein expression in 5 cSCC tumors relative to matched normal tissues (Fig. 1F). Cell line analysis further supported the significance of FSTL1, as A431, Colo16, and SCL1 human cSCC lines exhibited higher FSTL1 levels compared to normal human keratinocytes (HaCat) (Fig. 1G). Morphological distinctions between tumor and normal tissues were evident through H&E staining of paraffin sections (Fig. 1H). Immunohistochemistry validated the elevated FSTL1 expression in tumors compared to adjacent normal tissues (Fig. 1I). Cellular immunofluorescence localized FSTL1 predominantly to the cytosol of A431 cells (Fig. 1J). In summary, these preliminary findings collectively suggest an oncogenic role for FSTL1 in cSCC, supported by its elevated expression in both clinical samples and cell lines, indicating its potential significance in the context of cutaneous squamous cell carcinoma.

### FSTL1 predicts worse outcome in cSCC patients

**Fig. 2:**
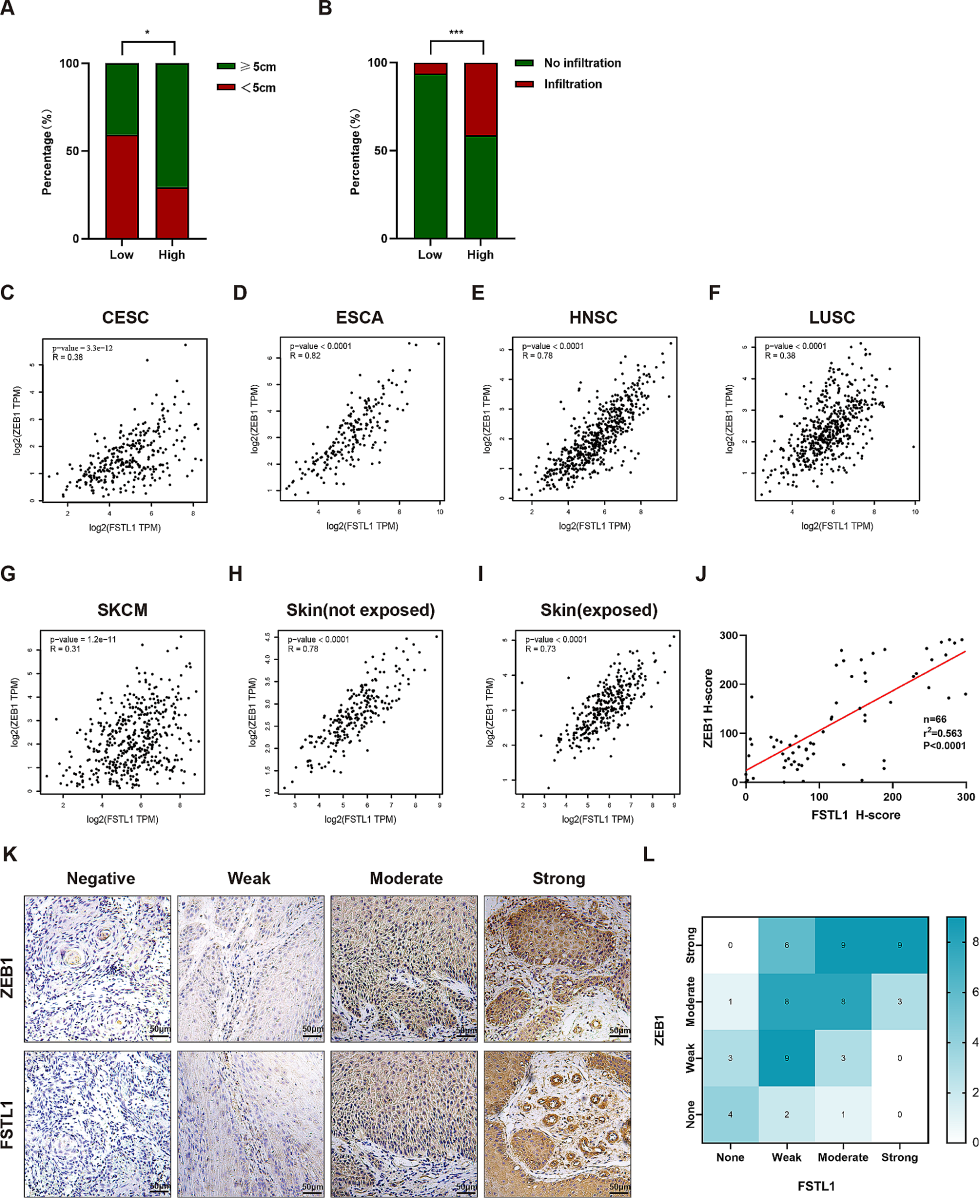
FSTL1 predicts worse outcome in cSCC patients. (**A**) Tumor size in high vs. low FSTL1 groups. (**B**) Local infiltration in high vs. low FSTL1 groups. (**C**–**I**) FSTL1 and ZEB1 expression correlation in CESC, ESCA, HNSC, LUSC, SKCM and skin (exposed/non-exposed). (**J**) FSTL1 and ZEB1 correlation in cSCC tissues. (**K**–**L**) FSTL1 and ZEB1 immunohistochemistry and heatmap in tissues, scale bars 50 μm. Data were presented as the mean ± SD. (ns, non-significant; *p < 0.05; **p < 0.01; ***p < 0.001; ****p < 0.0001)

To investigate the prognostic impact of FSTL1 in cSCC, immunohistochemistry was performed on paraffin sections from 66 cSCC patients. Based on immunohistochemical staining intensity and the percentage of positively stained area, samples were categorized into high or low FSTL1 expression groups. Chi-square test or Fisher exact test was employed to assess the association of FSTL1 expression with clinical parameters. The results revealed a significant correlation between high FSTL1 expression and larger tumor size, as well as local tissue infiltration (*p* < 0.05) (Table 1; Fig. 2A-B). Given the clinical relevance of these findings, we speculated that FSTL1 might be linked to tumor Epithelial-Mesenchymal Transition. To explore this, we analyzed the correlation between FSTL1 and the EMT marker ZEB1 expression in various squamous cancers, skin tumors, and sun-exposed/unexposed skin using data from TCGA and GTEx. Linear correlation and statistical significance (*p* < 0.05) between ZEB1 and FSTL1 were observed in cervical cancer (CESC), esophageal cancer (ESCA), head and neck cancer (HNSC), lung squamous cell carcinoma (LUSC), melanoma (SKCM), and sun-exposed/unexposed skin (Fig. 2C-I). Immunohistochemistry conducted on the 66 cSCC samples further confirmed a linear correlation between ZEB1 and FSTL1 expression (r2 = 0.563, *p* < 0.001) (Fig. 2J-L). Based on these observations, we hypothesize that FSTL1 is associated with a poor prognosis in cSCC.

**Table 1:** The relationship between FSTL1 expression and clinical parameters

Parameter		FSTL1 expression			
		*N* = 66	Low	High	P value
Age	≤54	33	12	21	0.084
	> 54	33	20	13	
Gender	Male	39	18	21	0.803
	Female	27	14	13	
Differentiation	Good	50	27	23	0.113
	Bad	16	5	11	
Lesion type	Ulceration	62	31	31	0.614
	Not ulceration	4	1	3	
Lesion size(cm)	< 5	32	19	13	0.025
	≥ 5	34	10	24	
Tumor embolus	Yes	1	0	1	> 0.999
	No	65	32	33	
Nerve invasion	Yes	2	1	1	> 0.999
	No	64	31	33	
Local infiltration	Yes	16	2	14	0.001
	No	50	30	20	
Lymph node involvement	Yes	4	1	3	0.614
	No	62	31	31	

Pearson’s chi-squared test or Fisher’s exact test

### FSTL1 promotes cell proliferation migration and invasion in vitro and in vivo

**Fig. 3:**
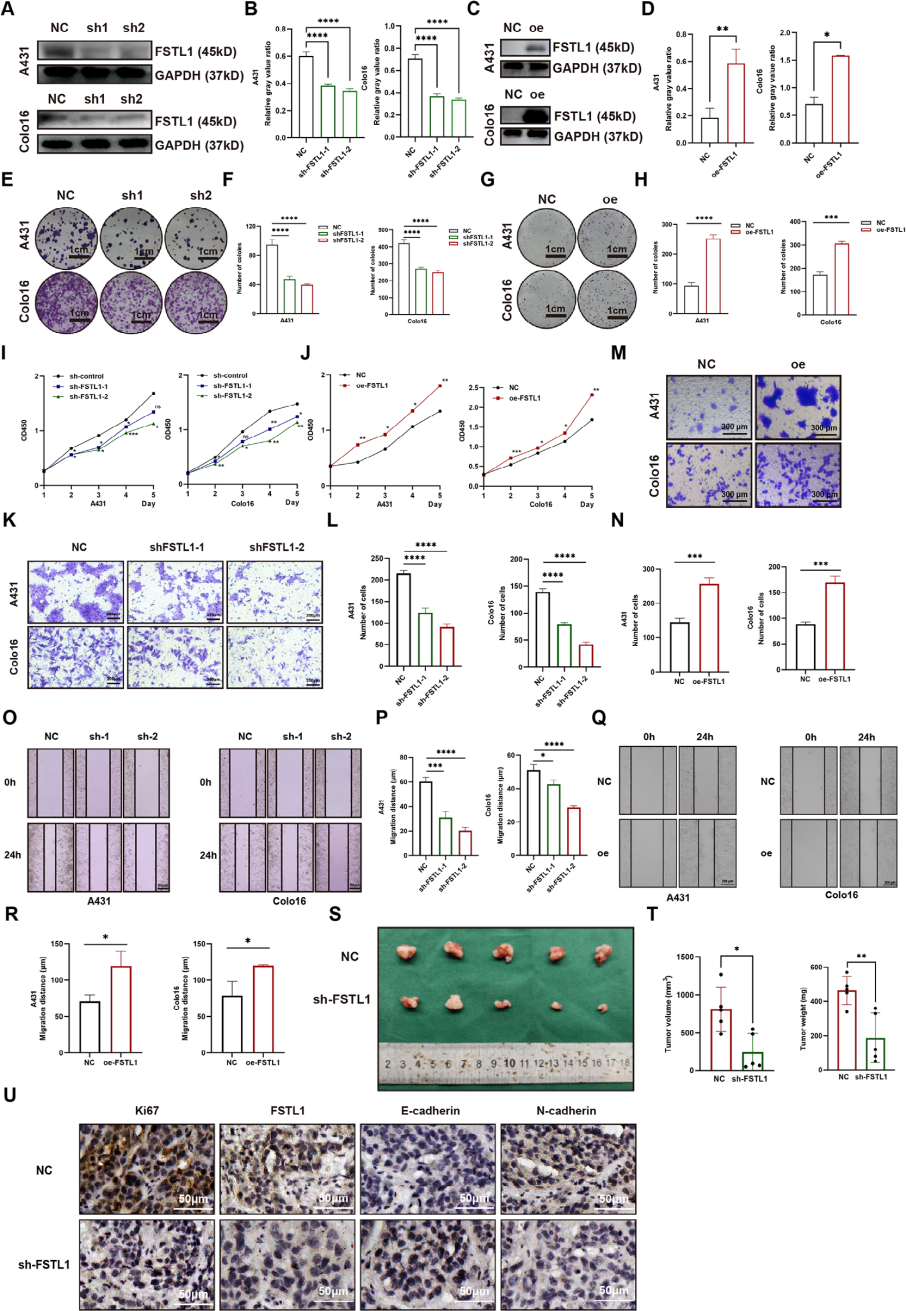
FSTL1 promotes proliferation and invasion in vitro and in vivo. (**A**–**B**) FSTL1 knockdown efficiency by western blot. (**C**–**D**) FSTL1 overexpression efficiency by western blot. (**E**–**F**) Clone formation after FSTL1 knockdown, scale bars 1 cm. (**G**–**H**) Clone formation after FSTL1 overexpression, scale bars 1 cm. (**I**–**J**) Cell viability by CCK8 assay. (**K**–**L**) Invasion by Transwell after FSTL1 knockdown, scale bars 200 μm. (**M**–**N**) Invasion by Transwell after FSTL1 overexpression, scale bars 300 μm. (**O**–**P**) Migration by scratch assay after FSTL1 knockdown, scale bars 50 μm. (**Q**–**R**) Migration by scratch assay after FSTL1 overexpression, scale bars 200 μm. (**S**–**T**) Tumor size, weight and volume. (**U**) IHC for Ki67, FSTL1, E-cadherin and N-cadherin, scale bars 50 μm. Data were presented as the mean ± SD. (ns, non-significant; *p < 0.05; **p < 0.01; ***p < 0.001; ****p < 0.0001)

To investigate the impact of FSTL1 on cSCC malignancy, we manipulated FSTL1 expression levels in A431 and Colo16 cells through lentiviral shRNAs or overexpression plasmids, ensuring efficient knockdown or overexpression as confirmed by western blot (Fig. 3A-D). Colony formation assays demonstrated a significant reduction in colony numbers in sh-FSTL1 cells compared to control cells (Fig. 3E-F). Conversely, the FSTL1 overexpression group exhibited a substantial increase in the number of clones formed compared to the control group (Fig. 3G-H). CCK8 assay, measuring the absorbance peaks at 450 nm to assess cellular viability, indicated weakened proliferative activity in the FSTL1 knockdown group and enhanced activity in the FSTL1 overexpression group (Fig. 3I-J). Migration and invasion abilities of cSCC cells were evaluated using scratch and matrix gel transwell assays. The results indicated a decrease in the number of cells crossing the matrix gel (Fig. 3K-L) and a shortened migration distance (Fig. 3O-P) in the FSTL1 knockdown group, suggesting weakened migration and invasion abilities. In contrast, the FSTL1 overexpression group exhibited an increase in the number of cells crossing the matrix gel (Fig. 3M-N) and a longer migration distance (Fig. 3Q-R), indicating enhanced migration and invasion abilities. We also conducted investigations into the effects of FSTL1 on apoptosis and the cell cycle. The results revealed that knocking down FSTL1 did not elicit significant changes in apoptosis when compared with the control group (Fig S1 A-B). Furthermore, there were no substantial alterations observed in the cell cycle following the knockdown of FSTL1(Fig S1 C-D). These findings suggest that FSTL1 may not play a prominent role in regulating apoptosis or influencing the progression of the cell cycle under the conditions tested in this study. In vivo experiments involved injecting sh-FSTL1 or control A431 cells into nude mice, revealing significantly smaller tumor volumes and weights in the sh-FSTL1 groups compared to controls (Fig. 3S-T). Paraffin sections of mouse tumor tissues were subjected to immunohistochemistry, showing weakened Ki67 staining (indicating reduced proliferation), increased E-cadherin (indicating more epithelial properties), and reduced N-cadherin (indicating less mesenchymal properties) in sh-FSTL1 tumors (Fig. 3U). Collectively, our results strongly indicate that suppressing FSTL1 expression mitigates both proliferation migration and invasion of cSCC, whereas heightened levels of FSTL1 have the opposite effect, promoting increased malignancy.

### FSTL1 interacts with ZEB1 and enhances EMT

**Fig. 4:**
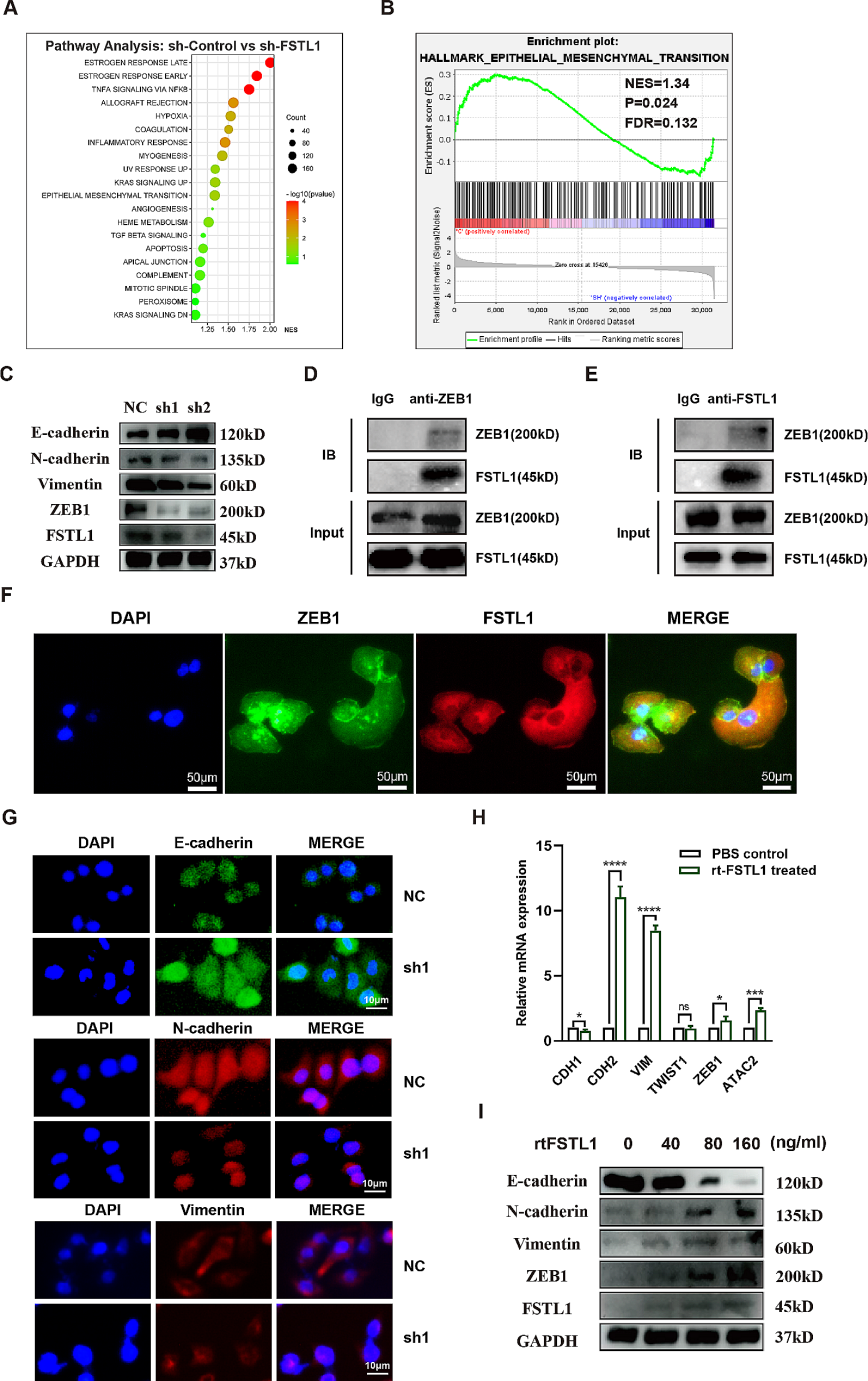
FSTL1 interacts with ZEB1 and enhances EMT. (**A**–**B**) Pathway enrichment analysis of FSTL1 knockdown. (**C**) Western blot of EMT markers after FSTL1 knockdown. (**D**–**E**) Co-IP for FSTL1 and ZEB1. (**F**) Immunofluorescence co-localization of FSTL1 and ZEB1, scale bars 50 μm. (**G**) Immunocytochemistry of E-cadherin, N-cadherin and Vimentin after FSTL1 knockdown, scale bars 10 μm. (**H**) qPCR after A431 rt-FSTL1 treatment. (**I**) Western blot after A431 rt-FSTL1 treatment. Data were presented as the mean ± SD. (ns, non-significant; *p < 0.05; **p < 0.01; ***p < 0.001; ****p < 0.0001)

To unveil the pathways influenced by FSTL1, we conducted an analysis on the GSE63329 GEO dataset, focusing on cSCC cells where FSTL1 expression was knocked down. Gene Set Enrichment Analysis (GSEA) of this dataset disclosed a correlation between FSTL1 expression and Epithelial-Mesenchymal Transition (Fig. 4A). Further examination through enrichment plots indicated that elevated FSTL1 levels promoted EMT, while FSTL1 knockdown hindered this process (Fig. 4B). Validation experiments carried out in A431 cells underscored these findings. FSTL1 knockdown resulted in decreased levels of ZEB1 and increased expression of E-cadherin, concurrently with reductions in N-cadherin and Vimentin, confirming the inhibitory effect on EMT (Fig. 4C). Co-immunoprecipitation studies in A431 cells demonstrated a direct interaction between FSTL1 and ZEB1 (Fig. 4D-E). Immunocytochemistry further supported this interaction by revealing co-localization of FSTL1 and ZEB1 (Fig. 4F). Additionally, FSTL1 knockdown correlated with increased E-cadherin and decreased N-cadherin and Vimentin fluorescence intensities, aligning with the results obtained from western blots (Fig. 4G). Upon treating A431 cells with recombinant FSTL1 (rt-FSTL1), we observed an elevation in ZEB1, CDH2, Vimentin, and ATAC2 mRNAs, coupled with a decrease in CDH1 mRNA (Fig. 4H). Furthermore, western blot analysis demonstrated a dose-dependent increase in ZEB1, N-cadherin, and Vimentin expression, accompanied by a reduction in E-cadherin expression, consistent with the mRNA expression patterns (Fig. 4I). Collectively, our data support the hypothesis that FSTL1 plays a critical role in influencing EMT in cSCC, particularly through its interaction with ZEB1. The comprehensive analysis of the GSE63329 dataset, combined with experimental validations in A431 cells, provides compelling evidence for the regulatory role of FSTL1 in the EMT process, shedding light on potential mechanisms underlying cSCC progression.

### THOC7-AS1 promotes cSCC progression and associates with OCT1 -FSTL1

**Fig. 5:**
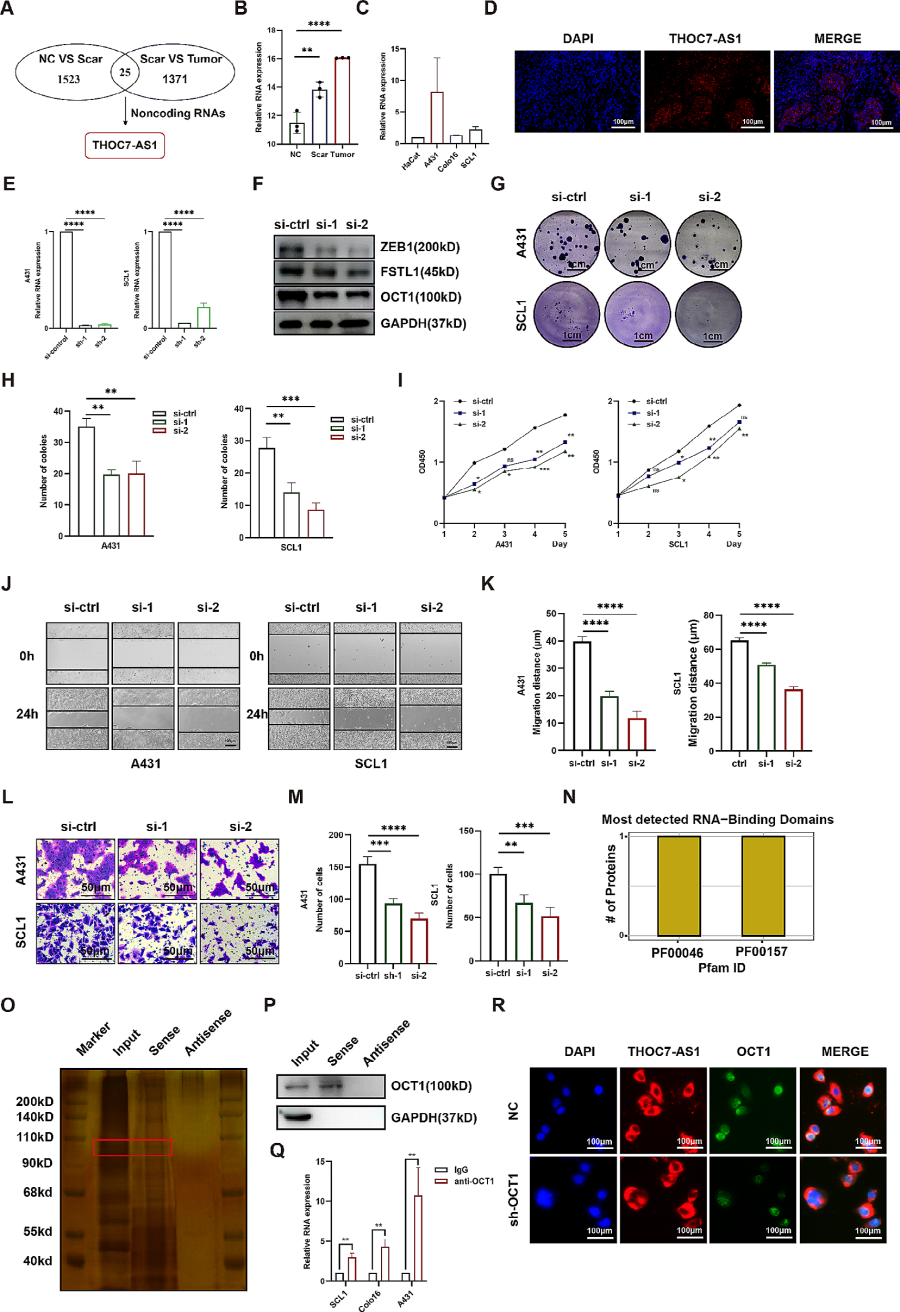
THOC7-AS1 promotes cSCC progression and associates with OCT1-FSTL1. (**A**) RNA-seq for key lncRNAs. (**B**) THOC7-AS1 expression in tissues. (**C**) THOC7-AS1 expression in cell lines. (**D**) THOC7-AS1 FISH in A431, scale bars 100 μm. (**E**) THOC7-AS1 knockdown efficiency. (**F**) OCT1, ZEB1, and FSTL1 levels after THOC7-AS1 knockdown. (**G**–**H**) Clone formation after THOC7-AS1 knockdown, scale bars 1 cm. (**I**) Cell viability after THOC7-AS1 knockdown. (**J**–**K**) Migration by scratch assay, scale bars 100 μm. (**L**–**M**) Invasion by Transwell, scale bars 50 μm. (**N**) THOC7-AS1 and OCT1 binding prediction. (**O**–**P**) Silver staining and western blot after RNA pull-down. (**Q**) THOC7-AS1 RNA levels after RIP. (**R**) THOC7-AS1 and OCT1 FISH co-localization, scale bars 100 μm. Data were presented as the mean ± SD. (ns, non-significant; *p < 0.05; **p < 0.01; ***p < 0.001; ****p < 0.0001)

To investigate potential upstream regulators of FSTL1 in cSCC, we conducted high-throughput sequencing on three distinct tissue samples (normal skin, precancerous scar, Marjolin ulcer) and identified 25 differentially expressed non-coding RNAs. Among them, the long non-coding RNA THOC7-AS1 emerged as a noteworthy candidate for further exploration (Fig. 5A). Expression analysis demonstrated a stepwise increase in THOC7-AS1 levels from normal to precancerous to tumor tissues, indicating its potential involvement in cSCC progression (Fig. 5B). THOC7-AS1 expression was also found to be elevated in A431, Colo16, and SCL1 cells compared to HaCat keratinocytes, reinforcing its relevance in cSCC cell lines (Fig. 5C). Further characterization through FISH assay on fresh cSCC samples revealed predominant cytoplasmic localization of THOC7-AS1, with higher expression levels in tumors compared to adjacent tissues (Fig. 5D). To investigate the functional impact of THOC7-AS1, we employed siRNAs for knockdown, resulting in reduced protein levels of both FSTL1 and ZEB1 (Fig. 5E-F). Functional assays demonstrated that THOC7-AS1 knockdown led to decreased clone numbers in A431 and SCL1 cells, as evidenced by clone formation assays (Fig. 5G-H), and a reduction in cell viability by CCK8 assays (Fig. 5I). Scratch assays revealed inhibited migration upon THOC7-AS1 knockdown (Fig. 5J-K), and Transwell assays demonstrated a decrease in piercing cell numbers with THOC7-AS1 interference (Fig. 5L-M). To elucidate the potential regulatory mechanism of THOC7-AS1 on FSTL1, bioinformatics analysis identified the intermediate molecule OCT1, indicating an interaction between THOC7-AS1 and OCT1 in specific domains (catRAPID omics v2.0) (Fig. 5N). RNA pull-down and RIP assays in A431 cells validated this interaction, demonstrating that THOC7-AS1 could pull down OCT1 and vice versa (Fig. 5O-Q). Additionally, FISH fluorescence double-staining revealed reduced co-localization of THOC7-AS1 and OCT1 upon OCT1 knockdown, further supporting their interaction (Fig. 5R). In conclusion, our findings suggest that THOC7-AS1 plays a role in promoting cSCC progression, potentially regulating FSTL1 through its interaction with OCT1.

### OCT1 binds to the FSTL1 promoter

**Fig. 6:**
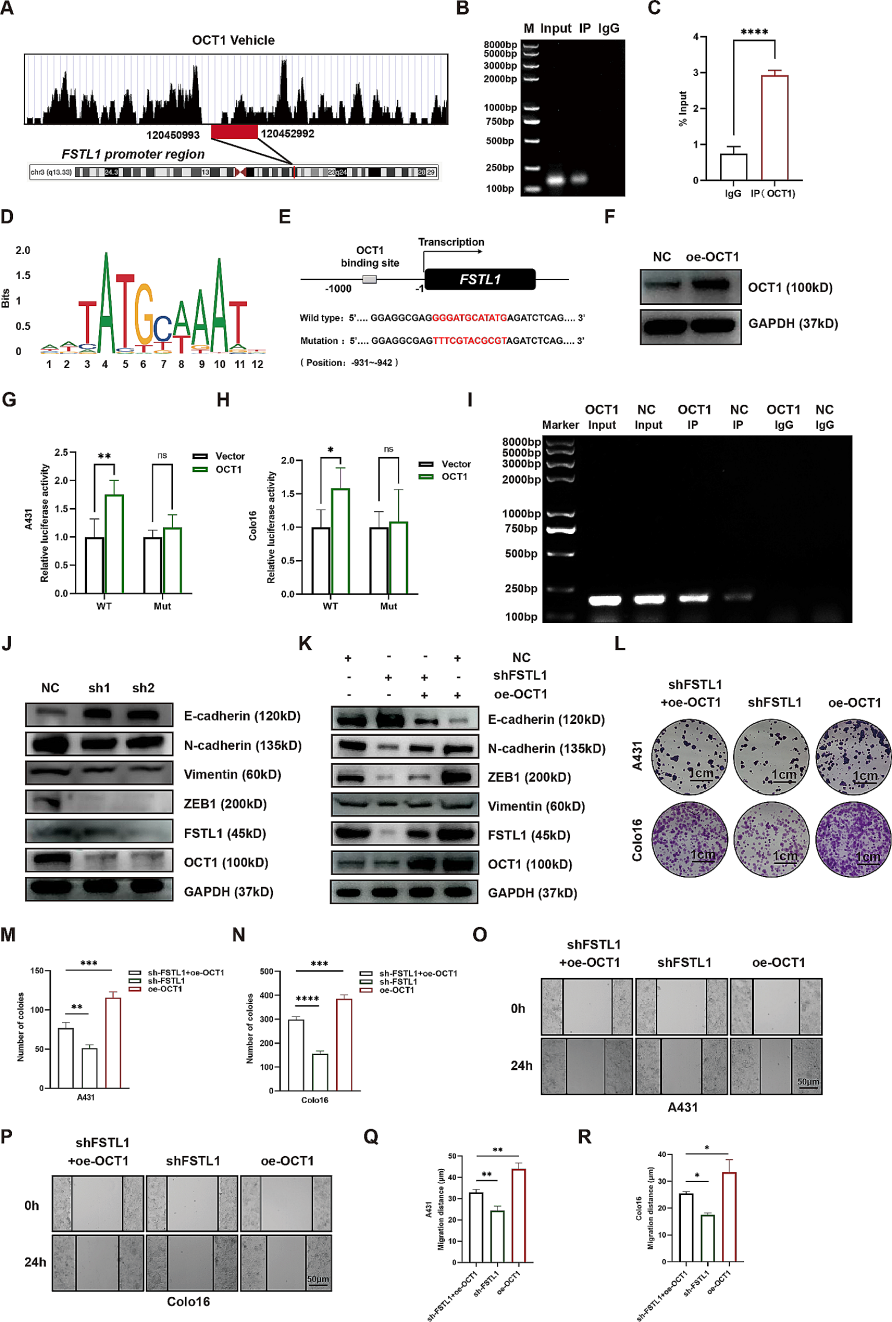
OCT1 binds to the FSTL1 promoter. (**A**) Peak map of OCT1 binding to the FSTL1 promoter. (**B**–**C**) OCT1 ChIP-agarose gel and qPCR showing OCT1 binding to the FSTL1 promoter. (**D**) Putative OCT1 motifs in the FSTL1 promoter by JASPAR. (**E**) Wild-type and mutant FSTL1 promoter sequences. (**F**) OCT1 overexpression verification by western blot. (**G**–**H**) Dual-luciferase reporter assay analyzing OCT1 binding to the FSTL1 promoter. (**I**) ChIP-agarose gel showing OCT1 binding to the FSTL1 promoter with OCT1 overexpression versus control. (**J**) Western blot of EMT markers after OCT1 knockdown. (**K**) Western blot of EMT markers after OCT1 overexpression rescue of FSTL1 knockdown. (**L**–**N**) Colony formation after OCT1 overexpression rescue, scale bars 1 cm. (**O**–**R**) Migration by scratch assay after OCT1 overexpression rescue, scale bars 50 μm. Data were presented as the mean ± SD. (ns, non-significant; *p < 0.05; **p < 0.01; ***p < 0.001; ****p < 0.0001)

Bioinformatic analyses using resources such as http://cistrome.org/db/#/ and https://genome.ucsc.edu/ predicted the presence of an OCT1 binding site within the FSTL1 promoter (Fig. 6A). Subsequently, our ChIP Agarose gel and qPCR assays confirmed the direct binding of OCT1 to the FSTL1 DNA fragment, providing experimental validation for the predicted binding site (Fig. 6B-C). Additionally, JASPER site prediction identified specific OCT1 binding motifs (Fig. 6D) and a putative FSTL1 binding site, further supporting the regulatory relationship (Supplementary Table S5). To functionally validate this interaction, we generated dual luciferase reporter constructs containing either wild-type or mutant FSTL1 promoter sequences in A431 and Colo16 cells (Fig. 6E). Co-transfection with an OCT1 overexpressing plasmid (Fig. 6F) revealed higher luciferase activity in the wild-type compared to the mutant reporter in both cell lines (Fig. 6G-H). Further supporting this, an agarose gel assay in A431 cells demonstrated increased OCT1 binding to the FSTL1 DNA in the OCT1 overexpression group (Fig. 6I). To explore the impact of OCT1 on tumor cell behavior, we conducted OCT1 knockdown experiments in A431 and Colo16 cells. Western blot results indicated that OCT1 knockdown led to decreased protein expression of FSTL1, ZEB1, N-cadherin, and Vimentin, accompanied by increased expression of E-cadherin (Fig. 6J). Moreover, OCT1 overexpression effectively rescued FSTL1 knockdown, restoring protein levels of FSTL1, ZEB1, N-cadherin, and Vimentin, while reducing E-cadherin compared to FSTL1 knockdown alone (Fig. 6K).

And functional assays demonstrated that OCT1 overexpression partially rescued the proliferative capacity (Fig. 6L-N) and migratory ability (Fig. 6O-R) attenuated by FSTL1 knockdown. In conclusion, our results indicate that OCT1 directly binds to the FSTL1 promoter, inducing its expression and thereby promoting cSCC proliferation and migration. These findings unveil a crucial regulatory axis involving OCT1 and FSTL1 in cSCC progression.

### THOC7-AS1 ASO diminishes cSCC progression

**Fig. 7:**
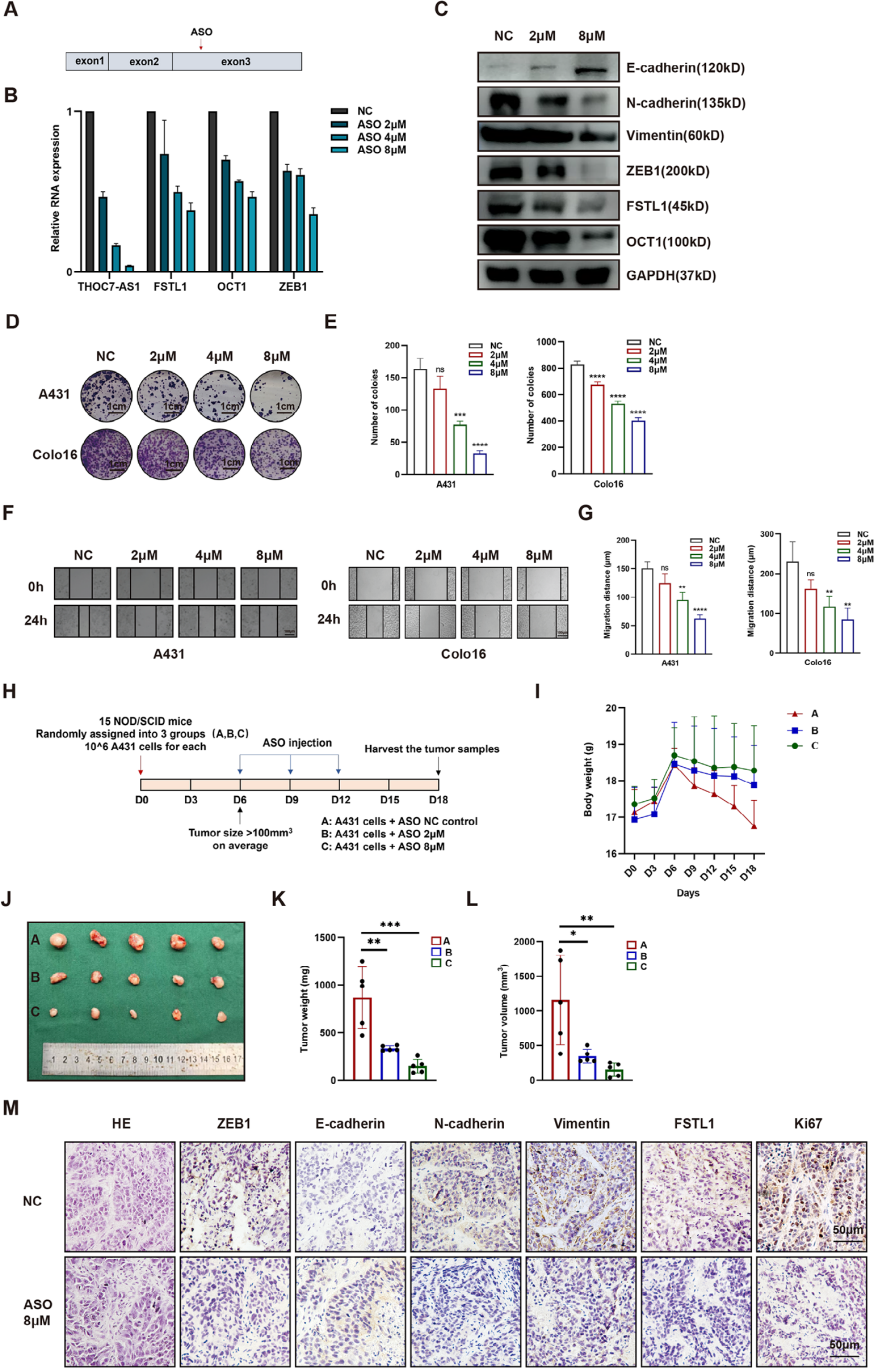
THOC7-AS1 ASO diminishes cSCC progression. (**A**) Schematic of ASO targets on THOC7-AS1. (**B**) THOC7-AS1, FSTL1, ZEB1, and OCT1 RNA expression after ASO treatment by qPCR in A431. (**C**) FSTL1, OCT1 and EMT-markers protein levels after ASO treatment. (**D**–**E**) Colony formation after ASO treatment. (**F**–**G**) Migration by scratch assay after ASO treatment. (**H**) Schematic of ASO treatment in xenograft model. (**I**) Mouse weight changes with ASO treatment. (**J**–**L**) Tumor size, weight, and volume with ASO treatment. (**M**) **H**&**E** and IHC for Ki67, FSTL1, ZEB1, E-cadherin, N-cadherin, and vimentin after ASO treatment, scale bars 100 μm. Data were presented as the mean ± SD. (ns, non-significant; *p < 0.05; **p < 0.01; ***p < 0.001; ****p < 0.0001)

The therapeutic potential of a THOC7-AS1 antisense oligonucleotide (ASO) was assessed in cSCC, employing a designed ASO targeting THOC7-AS1 (Fig. 7A). In A431 cells, treatment with the THOC7-AS1 ASO resulted in a dose-dependent reduction of THOC7-AS1 expression, with optimal inhibition observed at 8 µM (Fig. 7B). Furthermore, ASO treatment dose-dependently decreased the expression of FSTL1, OCT1, and ZEB1 at both mRNA and protein levels (Fig. 7B-C). Notably, proteins associated with the epithelial-mesenchymal transition (N-cadherin and Vimentin) were reduced, while E-cadherin, an epithelial marker, showed an increase. In vitro, the THOC7-AS1 ASO exhibited a dose-dependent inhibitory effect on A431 clone formation and migration, as demonstrated by reduced clone numbers and migration distance (Fig. 7D-G). Moving to in vivo experiments, A431-tumor bearing nude mice were subjected to control ASO, 2 µM ASO, or 8 µM ASO treatments (Fig. 7H). While mouse weights exhibited a decline after tumor formation, the weight loss was more moderate with ASO treatment (Fig. 7I). Importantly, ASO treatment resulted in a dose-dependent reduction in tumor weights and volumes compared to the control group (Fig. 7J-L). Immunohistochemistry of the tumor tissues revealed distinct alterations in protein expression patterns. ASO-treated tumors exhibited weakened staining for FSTL1, Ki67, ZEB1, N-cadherin, and Vimentin, along with increased E-cadherin staining, suggesting a favorable impact on proliferation and epithelial-mesenchymal transition markers (Fig. 7M). In conclusion, the THOC7-AS1 ASO effectively modulated the expression of FSTL1 and its downstream targets through the THOC7-AS1/OCT1/FSTL1 axis, leading to a significant inhibition of cSCC tumor growth. These results provide valuable insights into the potential therapeutic efficacy of targeting THOC7-AS1 in the context of cSCC.

## Discussion

The incidence of cSCC is rapidly rising, particularly in high-risk populations including organ transplant recipients, immunocompromised patients, and those with severe comorbidities [32]. In these groups, metastasis rates may reach 37%, with ∼90% occurring within 2 years of initial diagnosis. Over two-thirds of patients with metastatic cSCC die from locally invasive disease or lymph node involvement [33, 34]. These high-risk patients often cannot be treated with surgery or radiation and have poor responses to current drug and targeted therapies. Exploring new therapeutic targets is thus critical for cSCC management.

FSTL1 is a secreted glycoprotein known to regulate many diseases and is essential in embryonic development [35]. As described in precancerous lesions of liver cancer, FSTL1 can remodel macrophage function by modulating PKM2 to promote liver fibrosis [36]. In the field of oncology research, studies have shown that FSTL1 plays an important role in promoting tumor progression in a variety of tumors, including hepatocellular carcinoma, gastric cancer, colorectal cancer, osteosarcoma and nasopharyngeal carcinoma [7, 9, 37–39]. For instance, FSTL1 can bind to TLR4, activating AKT/mTOR/4EBP1 signaling and enhancing hepatocellular carcinoma metastasis and stemness [9]. Besides, TGFβ1-induced FSTL1 binding to Vimentin activates focal adhesion signaling, promoting colorectal cancer cell migration, invasion, and metastasis [37]. Additionally, FSTL1 knockdown in osteosarcoma inhibits proliferation, invasion, spheroid formation, and ALCAM expression, while anti-FSTL1 treatment restores NK cell activity, killing tumor cells and inhibiting osteosarcoma growth and metastasis [38]. Moreover, in nasopharyngeal carcinoma, FSTL1 blocks Wnt7a inhibition of ERK phosphorylation, inducing MMP9 production, extracellular matrix degradation, and metastasis [39]. However, FSTL1’s role in skin tumors is unknown. Here, we demonstrate for the first time that FSTL1 is an oncogene in cSCC, with elevated expression in tissues and cells correlating with poor prognostic features like tumor size and local infiltration. Meanwhile, in our study, we observed that knocking down FSTL1 expression significantly inhibits cSCC cell viability, proliferation, migration, and invasion in vitro and in vivo. This implies that FSTL1 promotes malignant biological behaviors in cSCC.

Mounting evidence indicates FSTL1 plays a key role in tumor formation [40–42]. However, its specific mechanisms regulating cSCC progression remain controversial. Based on immunohistochemical data from clinical samples, we have observed that high expression of FSTL1 is closely associated with local tumor infiltration. Thus, we hypothesized that FSTL1 promotes tumor infiltration through EMT. We subsequently analyzed the relationship between FSTL1 and ZEB1 in other squamous carcinomas in the TCGA and GTEx data, and the data show that FSTL1 and ZEB1 expression are linearly correlated in these tumors. To verify FSTL1’s pro-tumorigenic molecular actions through EMT in cSCC, we found by RNA-seq pathway analysis that FSTL1 expression strongly impacts EMT. This was validated by immunoprecipitation, immunofluorescence co-localization, and western blotting, demonstrating FSTL1’s interaction with ZEB1. Our experiments showed that FSTL1 knockdown enhances E-cadherin expression while suppressing ZEB1, N-cadherin and Vimentin expression, inhibiting EMT.

We next explored upstream regulators of FSTL1 overexpression in cSCC. Bioinformatic analysis showed that the transcription factor OCT1 could bind to FSTL1 promoter. Our ChIP and dual luciferase reporter assays confirmed OCT1 binds the FSTL1 promoter to induce its expression in cSCC cells. As an essential transcription factor, OCT1 plays a crucial role in proliferation, metastasis and metabolism [43–45]. For instance, study has showed that OCT1 binds the ALDOA promoter, enhancing activity and metabolic reprogramming to drive progression and drug resistance in colon cancer [43]. In prostate cancer, OCT1 binds and upregulates PFN2 to stimulate tumor growth [44]. Takuya also found that OCT1 is a poor prognostic factor for breast cancer patients and promotes cell proliferation via inducing NCAPH [45]. Consistent with the above results, we found OCT1 binds to FSTL1 promoter and promotes FSTL1 expression. Besides, we discovered that knockdown of OCT1 inhibits the expression of EMT-related proteins such as ZEB1 in cSCC cell lines. Moreover, OCT1 overexpression also partially rescues proliferative capacity and migratory ability attenuated by FSTL1 knockdown. Our results showed that OCT1 regulates FSTL1 expression and enhances aberrant EMT activation, facilitating cSCC growth.

The interplay between lncRNAs and transcription factors is crucial for tumorigenesis [46–49]. For example, in gastric cancer, DLX6-AS1 acts as a ceRNA for miR-204-5p, upregulating OCT1. This DLX6-AS1/miR-204-5p/OCT1 feedback loop enhances EMT and progression [50]. A recent study also revealed that lncRNA AC026401.3 interacts with OCT1 to intensify sorafenib and lenvatinib resistance by activating E2F2 signaling in hepatocellular carcinoma [51]. Similarly, through RNA-seq, we identified the lncRNA THOC7-AS1 as highly expressed in cSCC. Bioinformatics analysis revealed that THOC7-AS1 and OCT1 binds to each other. This was verified by our RNA pull-down, RIP, and FISH immunofluorescence assays. We also demonstrated THOC7-AS1/OCT1/FSTL1 axis promotes EMT and cSCC progression using knockdowns, overexpression plasmid and rescuing functional assays. Finally, antisense oligonucleotides of THOC7-AS1 were designed and synthesized. Preliminary tests of the ASO showed significant anti-tumor effects in vitro and in vivo, highlighting the importance of this axis in cSCC.

Our study provides several advantages in the diagnosis and treatment of cutaneous squamous cell carcinoma. Firstly, we observed a significant upregulation of FSTL1 expression in both cell lines and tissues compared to the control group. The abnormal elevation of FSTL1 in tumors is closely associated with poor prognosis, suggesting its potential as a biomarker for cSCC. This finding contributes to the assessment of FSTL1 as a potential biomarker, facilitating more accurate diagnosis and prediction of disease progression. Secondly, the discovery of the FSTL1 as a novel target opens new avenues, potentially reducing the cytotoxic side effects associated with traditional drugs used in the treatment of advanced cSCC. However, the evaluation of drug side effects reduction and enhanced patient responsiveness remains to be assessed. Thirdly, we elucidated the molecular mechanism of FSTL1 regulation, demonstrating that the THOC7-AS1/OCT1/FSTL1 axis promotes epithelial-mesenchymal transition in cSCC cells, thus advancing tumor progression. Targeting this axis could be a multi-pronged approach to inhibit the progression of cSCC. Notably, the pharmaceutical company has registered the FSTL1-targeting drug PFI-103, exploring its role in osteosarcoma and solid tumors, providing a strong reference for further research.

In our study, conventional experimental and analytical methods were employed. Key molecular FSTL1 in cSCC was selected based on public databases, and its aberrant upregulation in cSCC was validated using qPCR, western blot, IHC, and ICC in both cell lines and tissue samples. Clinical samples analysis revealed a correlation between high FSTL1 expression and adverse parameters such as tumor size and local infiltration in cSCC patients, confirming its role as a pro-oncogenic molecule. Functionally, various experiments including cell transfection, clone formation, CCK8 assays, scratch assays, transwell assays, flow cytometry for apoptosis and cell cycle, and xenograft tumor formation were conducted to further demonstrate FSTL1’s role in promoting cell proliferation, migration, and invasion in cSCC. Molecularly, we utilized GSEA, qPCR, western blot, co-IP, ICC, RNAseq, FISH, RIP, RNA pull down, ChIP, dual-luciferase reporter gene assay, ASO, and cell function recovery experiments to clarify the regulation of FSTL1 by THOC7-AS1/OCT1 and its promotion of cSCC EMT. These conventional methods ensure the reproducibility and reliability of our research.

Our study first identified the pro-oncogenic role of FSTL1 in cSCC and revealed a novel mechanism, THOC7-AS1/OCT1/FSTL1, regulating EMT and promoting tumor progression, demonstrating significant innovation. Targeted therapies against FSTL1 or the THOC7-AS1/OCT1/FSTL1 axis may offer promising outcomes for late-stage cSCC patients. These targeted treatments, either alone or in combination with existing cytotoxic drug therapies, have the potential to improve treatment efficacy, mitigate drug side effects, and provide additional therapeutic options. However, further assessment is required to evaluate their effectiveness and side effects.

To enhance the effectiveness of targeted therapy, the utilization of green nanosilver particles provides a promising option. Research indicates that AgNPs synthesized using biological resources exhibit biocompatibility and possess strong antimicrobial, antioxidant, anti-inflammatory, and anti-tumor properties [52–55]. These nanoparticles have applications in diagnostics, cell labeling, biomarkers, drug delivery, cancer treatment, and water purification [54]. For instance, AgNPs obtained through green synthesis exhibit anti-cancer effects on various cancer cell lines, with inhibitory rates ranging from 25 to 81% at a concentration of 25 µg/mL [55]. Leveraging these properties, our study suggests that the FSTL1-targeting drug or ASO for THOC7-AS1/OCT1/FSTL1 axis could be labeled or delivered using nanosilver particles, which may enhance their anti-cancer effects.

In summary, our study reveals the THOC7-AS1/OCT1/FSTL1 axis as a key driver of cSCC proliferation, migration, and invasion. Targeting this axis may provide effective therapies.

### Electronic supplementary material

Below is the link to the electronic supplementary material.

Supplementary Material 1

Supplementary Material 2

Supplementary Material 3

Supplementary Material 4

Supplementary Material 5

Supplementary Material 6

Supplementary Material 7

## Data Availability

The data utilized and/or analyzed during this study are available from the corresponding author upon reasonable request.

## Abbreviations

THOC7-AS1: THO Complex Subunit 7 Antisense RNA 1
OCT1: Octamer-Binding Transcription Factor 1
FSTL1: Follistatin Like 1
EMT: Epithelial to Mesenchymal Transition
cSCC: Cutaneous Squamous Cell Carcinoma
qPCR: Quantitative Real-Time Polymerase Chain Reaction
IHC: Immunohistochemistry
IF: Immunofluorescence
ICC: Immunocytochemistry
IP: Immunoprecipitation
FISH: Fluorescence in Situ Hybridization
RIP: RNA Binding Protein Immunoprecipitation
ChIP: Chromatin Immunoprecipitation
ZEB1: Zinc Finger E-box Binding Homeobox 1
CCK8: Cell Counting Kit 8
GSEA: Gene Set Enrichment Analysis
ASO: antisense oligonucleotide
