# Supplementary material for: THOC7-AS1/OCT1/FSTL1 axis promotes EMT and serves as a therapeutic target in cutaneous squamous cell carcinoma
Source: J Transl Med. 2024 Apr 11;22:347. doi: 10.1186/s12967-024-05116-8 (PMC11010364; doi:10.1186/s12967-024-05116-8)
Supplement: Supplementary file 1 — Supplementary Material 1 [file 12967_2024_5116_MOESM1_ESM.docx]

**Table S2**

Primer sequences

| GAPDH F | CTGGGCTACACTGAGCACC |
| --- | --- |
| GAPDH R | AAGTGGTCGTTGAGGGCAATG |
| RPS18 F | GCGGCGGAAAATAGCCTTTG |
| RPS18 R | GATCACACGTTCCACCTCATC |
| FSTL1 F | CTCTAAAGGCAGCAACTACAGTG |
| FSTL1 R | CCCCTAAGCAACTTGTTGTTCTC |
| CDH1 F | CGAGAGCTACACGTTCACGG |
| CDH1 R | GGGTGTCGAGGGAAAAATAGG |
| CDH2 F | TGCGGTACAGTGTAACTGGG |
| CDH2 R | GAAACCGGGCTATCTGCTCG |
| Vimentin F | AGTCCACTGAGTACCGGAGAC |
| Vimentin R | CATTTCACGCATCTGGCGTTC |
| TWIST1 F | GTCCGCAGTCTTACGAGGAG |
| TWIST1 R | GCTTGAGGGTCTGAATCTTGCT |
| ZEB1 F | GATGATGAATGCGAGTCAGATGC |
| ZEB1 R | ACAGCAGTGTCTTGTTGTTGT |
| ATAC2 F | GTGTTGCCCCTGAAGAGCAT |
| ATAC2 R | GCTGGGACATTGAAAGTCTCA |
| THOC7-AS1 F | GAGGATCACCCCACTTCTGAC |
| THOC7-AS1 R | ACCCACCTCAAAAATCCCCT |
| POU2F1 F | ATGAACAATCCGTCAGAAACCAG |
| POU2F1 R | GATGGAGATGTCCAAGGAAAGC |
| SNAI1 F | TCGGAAGCCTAACTACAGCGA |
| SNAI1 R | AGATGAGCATTGGCAGCGAG |
| LINC01995 F | GATGGAAGGGACAGCCTTGA |
| LINC01995 R | AGAAACTCTGGTCCGAAGCC |

F: forward; R: reverse
