# Supplementary figures and images for: THOC7-AS1/OCT1/FSTL1 axis promotes EMT and serves as a therapeutic target in cutaneous squamous cell carcinoma
Source: J Transl Med. 2024 Apr 11;22:347. doi: 10.1186/s12967-024-05116-8 (PMC11010364; doi:10.1186/s12967-024-05116-8)

**Table S5**

Putative FSTL1 binding site for OCT1 motif


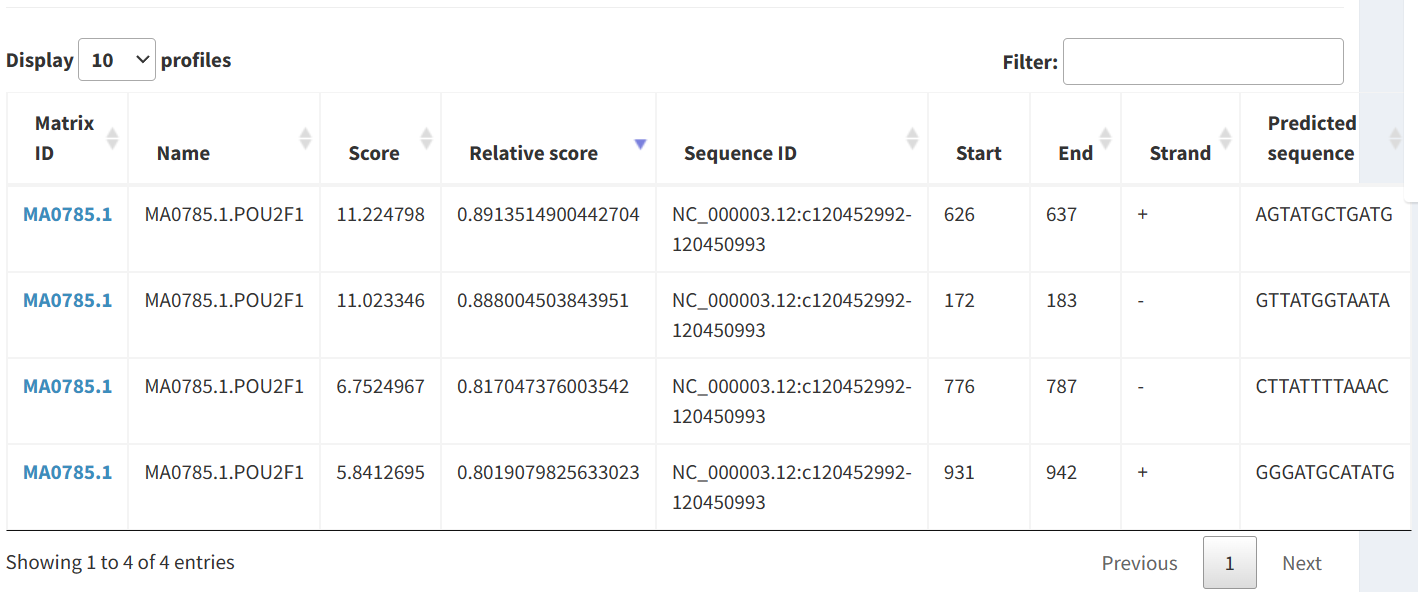

Supplement: Supplementary file 2 — Supplementary Material 2 [file 12967_2024_5116_MOESM2_ESM.docx]

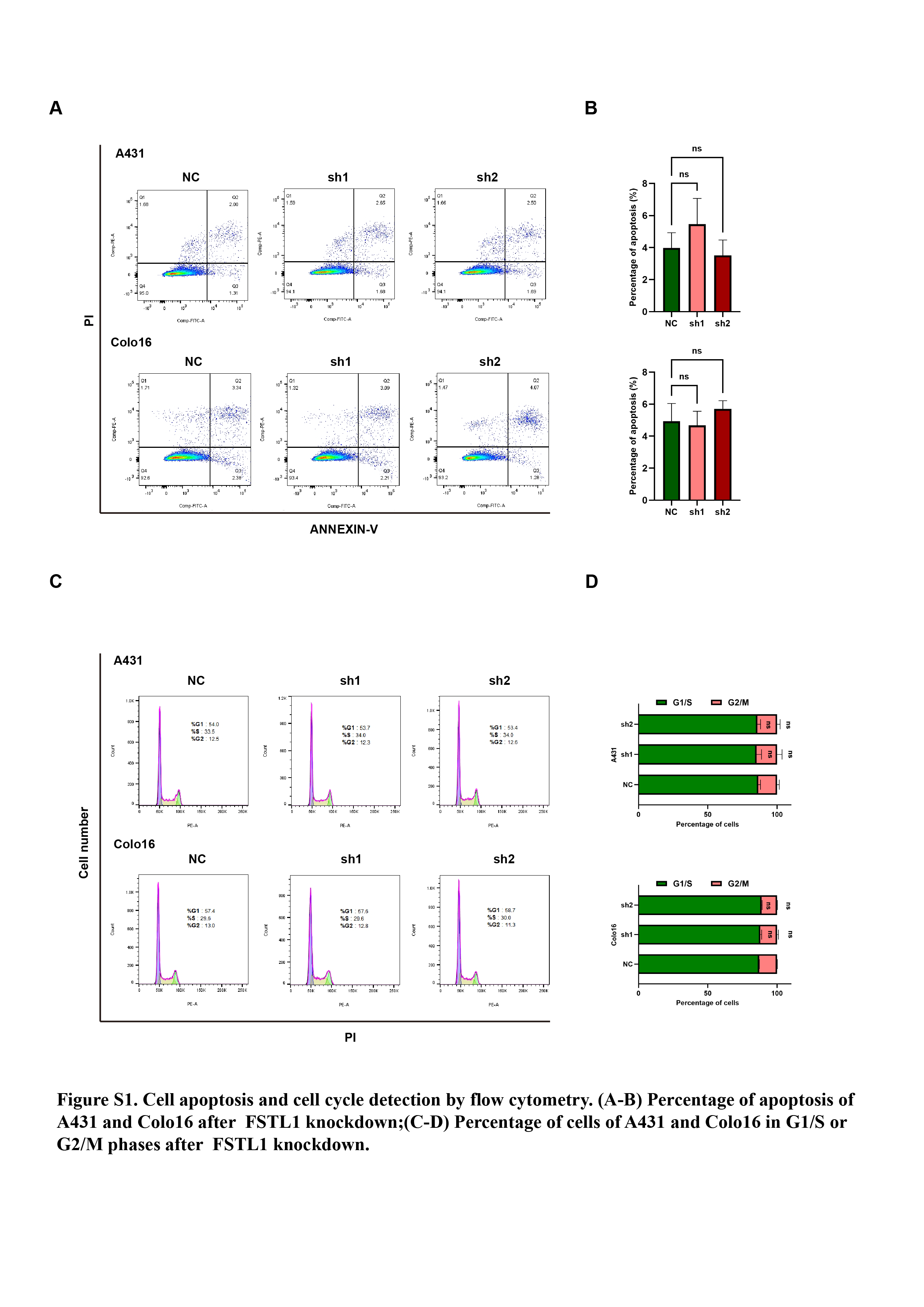

Supplement: Supplementary file 7 — Supplementary Material 7 [file 12967_2024_5116_MOESM7_ESM.png]
