# Supplementary material for: THOC7-AS1/OCT1/FSTL1 axis promotes EMT and serves as a therapeutic target in cutaneous squamous cell carcinoma
Source: J Transl Med. 2024 Apr 11;22:347. doi: 10.1186/s12967-024-05116-8 (PMC11010364; doi:10.1186/s12967-024-05116-8)
Supplement: Supplementary file 3 — Supplementary Material 3 [file 12967_2024_5116_MOESM3_ESM.docx]

**Table S1**

Antibody information:

| GAPDH | Servicebio, Wuhan, China |
| --- | --- |
| FSTL1 | Proteintech, Wuhan, China |
| ZEB1 | Proteintech, Wuhan, China |
| E-cadherin | Cell Signaling Technology, Boston, USA |
| N-cadherin | Proteintech, Wuhan, China |
| Vimentin | Proteintech, Wuhan, China |
| OCT1 | Proteintech, Wuhan, China |
| IgG | Servicebio, Wuhan, China |
| CoraLite594‑conjugated Goat Anti‑Rabbit IgG (H+L) | Proteintech, Wuhan, China |
| CoraLite488-conjugated Goat Anti-Rabbit IgG(H+L) | Proteintech, Wuhan, China |
