# Supplementary material for: THOC7-AS1/OCT1/FSTL1 axis promotes EMT and serves as a therapeutic target in cutaneous squamous cell carcinoma
Source: J Transl Med. 2024 Apr 11;22:347. doi: 10.1186/s12967-024-05116-8 (PMC11010364; doi:10.1186/s12967-024-05116-8)
Supplement: Supplementary file 4 — Supplementary Material 4 [file 12967_2024_5116_MOESM4_ESM.docx]

**Table S3**

Sequences of siRNAs

| siRNA-THOC7-AS1-1 | Sense strand | CGTATTAAGGGTGACAGCAGCAATA |
| --- | --- | --- |
|  | Antisense strand | TATTGCTGCTGTCACCCTTAATACG |
| siRNA-THOC7-AS1-2 | Sense strand | CAGAAGGTCAACTACTTCTAGGAAA |
|  | Antisense strand | TTTCCTAGAAGTAGTTGACCTTCTG |
| Negative control siRNA | Sense strand | UUCUCCGAACGUGUCACGU(dT)(dT) |
|  | Antisense strand | ACGUGACACGUUCGGAGAAdTdT |

Sequences of shRNA

| sh-FSTL1-1 | GCTAAGGAGCAAATCCAAGAT |
| --- | --- |
| sh-FSTL1-2 | CCAGGTTGATTACGATGGACA |
| sh-FSTL1-3 | GCCATCAATATTACAACGTAT |
| sh-FSTL1-4 | AGGCCTGTGTGTGGCAGTAAT |
| sh-POU2F1-1 | CCAAACTACCATCTCTCGATT |
| sh-POU2F1-2 | GCTGTGACGAATCTTTCAGTT |
| sh-POU2F1-3 | CCAGTCAACACCAAAGCGAAT |
| sh-POU2F1-4 | GCAACTGGGAACCTGGTATTT |
| NC | CCGGGGTTCTCCGAACGTGTCACGTCTCGAGACGTGACACGTTCGGAGAACCTTTTTG |

Sequences of overexpression plasmids

FSTL1:

ATGTGGAAACGCTGGCTCGCGCTCGCGCTCGCGCTGGTGGCGGTCGCCTGGGTCCGCGCCGAGGAAGAGCTAAGGAGCAAATCCAAGATCTGTGCCAATGTGTTTTGTGGAGCCGGCCGGGAATGTGCAGTCACAGAGAAAGGGGAACCCACCTGTCTCTGCATTGAGCAATGCAAACCTCACAAGAGGCCTGTGTGTGGCAGTAATGGCAAGACCTACCTCAACCACTGTGAACTGCATCGAGATGCCTGCCTCACTGGATCCAAAATCCAGGTTGATTACGATGGACACTGCAAAGAGAAGAAATCCGTAAGTCCATCTGCCAGCCCAGTTGTTTGCTATCAGTCCAACCGTGATGAGCTCCGACGTCGCATCATCCAGTGGCTGGAAGCTGAGATCATTCCAGATGGCTGGTTCTCTAAAGGCAGCAACTACAGTGAAATCCTAGACAAGTATTTTAAGAACTTTGATAATGGTGATTCTCGCCTGGACTCCAGTGAATTCCTGAAGTTTGTGGAACAGAATGAAACTGCCATCAATATTACAACGTATCCAGACCAGGAGAACAACAAGTTGCTTAGGGGACTCTGTGTTGATGCTCTCATTGAACTGTCTGATGAAAATGCTGATTGGAAACTCAGCTTCCAAGAGTTTCTCAAGTGCCTCAACCCATCTTTCAACCCTCCTGAGAAGAAGTGTGCCCTGGAGGATGAAACGTATGCAGATGGAGCTGAGACCGAGGTGGACTGTAACCGCTGTGTCTGTGCCTGTGGAAATTGGGTCTGTACAGCCATGACCTGTGACGGAAAGAATCAGAAGGGGGCCCAGACCCAGACAGAGGAGGAGATGACCAGATATGTCCAGGAGCTCCAAAAGCATCAGGAAACAGCTGAAAAGACCAAGAGAGTGAGCACCAAAGAGATCTAAOCT1:

ATGGCGGACGGAGGAGCAGCGAGTCAAGATGAGAGTTCAGCCGCGGCGGCAGCAGCAGCAGACTCAAGAATGAACAATCCGTCAGAAACCAGTAAACCATCTATGGAGAGTGGAGATGGCAACACAGGCACACAAACCAATGGTCTGGACTTTCAGAAGCAGCCTGTGCCTGTAGGAGGAGCAATCTCAACAGCCCAGGCGCAGGCTTTCCTTGGACATCTCCATCAGGTCCAACTCGCTGGAACAAGTTTACAGGCTGCTGCTCAGTCTTTAAATGTACAGTCTAAATCTAATGAAGAATCGGGGGATTCGCAGCAGCCAAGCCAGCCTTCCCAGCAGCCTTCAGTGCAGGCAGCCATTCCCCAGACCCAGCTTATGCTAGCTGGAGGACAGATAACTGGGCTTACTTTGACGCCTGCCCAGCAACAGTTACTACTCCAGCAGGCACAGGCACAGGCACAGCTGCTGGCTGCTGCAGTGCAGCAGCACTCCGCCAGCCAGCAGCACAGTGCTGCTGGAGCCACCATCTCCGCCTCTGCTGCCACGCCCATGACGCAGATCCCCCTGTCTCAGCCCATACAGATCGCACAGGATCTTCAACAACTGCAACAGCTTCAACAGCAGAATCTCAACCTGCAACAGTTTGTGTTGGTGCATCCAACCACCAATTTGCAGCCAGCGCAGTTTATCATCTCACAGACGCCCCAGGGCCAGCAGGGTCTCCTGCAAGCGCAAAATCTTCTAACGCAACTACCTCAGCAAAGCCAAGCCAACCTCCTACAGTCGCAGCCAAGCATCACCCTCACCTCCCAGCCAGCAACCCCAACACGCACAATAGCAGCAACCCCAATTCAGACACTTCCACAGAGCCAGTCAACACCAAAGCGAATTGATACTCCCAGCTTGGAGGAGCCCAGTGACCTTGAGGAGCTTGAGCAGTTTGCCAAGACCTTCAAACAAAGACGAATCAAACTTGGATTCACTCAGGGTGATGTTGGGCTCGCTATGGGGAAACTATATGGAAATGACTTCAGCCAAACTACCATCTCTCGATTTGAAGCCTTGAACCTCAGCTTTAAGAACATGTGCAAGTTGAAGCCACTTTTAGAGAAGTGGCTAAATGATGCAGAGAACCTCTCATCTGATTCGTCCCTCTCCAGCCCAAGTGCCCTGAATTCTCCAGGAATTGAGGGCTTGAGCCGTAGGAGGAAGAAACGCACCAGCATAGAGACCAACATCCGTGTGGCCTTAGAGAAGAGTTTCTTGGAGAATCAAAAGCCTACCTCGGAAGAGATCACTATGATTGCTGATCAGCTCAATATGGAAAAAGAGGTGATTCGTGTTTGGTTCTGTAACCGCCGCCAGAAAGAAAAAAGAATCAACCCACCAAGCAGTGGTGGGACCAGCAGCTCACCTATTAAAGCAATTTTCCCCAGCCCAACTTCACTGGTGGCGACCACACCAAGCCTTGTGACTAGCAGTGCAGCAACTACCCTCACAGTCAGCCCTGTCCTCCCTCTGACCAGTGCTGCTGTGACGAATCTTTCAGTTACAGGCACTTCAGACACCACCTCCAACAACACAGCAACCGTGATTTCCACAGCGCCTCCAGCTTCCTCAGCAGTCACGTCCCCCTCTCTGAGTCCCTCCCCTTCTGCCTCAGCCTCCACCTCCGAGGCATCCAGTGCCAGTGAGACCAGCACAACACAGACCACCTCCACTCCTTTGTCCTCCCCTCTTGGGACCAGCCAGGTGATGGTGACAGCATCAGGTTTGCAAACAGCAGCAGCTGCTGCCCTTCAAGGAGCTGCACAGTTGCCAGCAAATGCCAGTCTTGCTGCCATGGCAGCTGCTGCAGGACTAAACCCAAGCCTGATGGCACCCTCACAGTTTGCGGCTGGAGGTGCCTTACTCAGTCTGAATCCAGGGACCCTGAGCGGTGCTCTCAGCCCAGCTCTAATGAGCAACAGTACACTGGCAACTATTCAAGCTCTTGCTTCTGGTGGCTCTCTTCCAATAACATCACTTGATGCAACTGGGAACCTGGTATTTGCCAATGCGGGAGGAGCCCCCAACATCGTGACTGCCCCTCTGTTCCTGAACCCTCAGAACCTCTCTCTGCTCACCAGCAACCCTGTTAGCTTGGTCTCTGCCGCCGCAGCATCTGCAGGGAACTCTGCACCTGTAGCCAGCCTTCACGCCACCTCCACCTCTGCTGAGTCCATCCAGAACTCTCTCTTCACAGTGGCCTCTGCCAGCGGGGCTGCGTCCACCACCACCACCGCCTCCAAGGCACAGGATTACAAGGACGACGATGACAAGTGA
