# Supplementary material for: THOC7-AS1/OCT1/FSTL1 axis promotes EMT and serves as a therapeutic target in cutaneous squamous cell carcinoma
Source: J Transl Med. 2024 Apr 11;22:347. doi: 10.1186/s12967-024-05116-8 (PMC11010364; doi:10.1186/s12967-024-05116-8)
Supplement: Supplementary file 5 — Supplementary Material 5 [file 12967_2024_5116_MOESM5_ESM.docx]

**Table S4**

Primers of FSTL1 promoter or negative control for ChIP assay

| **FSTL1 Forward 1** | ACAACAGTGGGCACTCAACA |
| --- | --- |
| **FSTL1 Reverse 1** | GCCCACCCTGTCCATCTAAC |
| **FSTL1 Forward 2** | ACAAACCTTGGCAGCAGAGT |
| **FSTL1 Reverse 2** | GCCCACCCTGTCCATCTAAC |
| **FSTL1 Forward 3** | AGACTACAGGGGAGAGCCTG |
| **FSTL1 Reverse 3** | TTTTCTCAGAGCCGGCTTGT |
| **FSTL1 Forward 4** | AGACTACAGGGGAGAGCCTG |
| **FSTL1 Reverse 4** | CAGCTTCAGGGCCTGCTTAT |
| **FSTL1 Forward 5** | GCCCACCAAAACCAAGATGG |
| **FSTL1 Reverse 5** | TTTTCCAGGAAAGGGGTGGG |
| **GAPDH Forward** | TACTAGCGGTTTTACGGGCG |
| **GAPDH Reverse** | TCGAACAGGAGGAGCAGAGAGCGA |
