# Supplementary material for: THOC7-AS1/OCT1/FSTL1 axis promotes EMT and serves as a therapeutic target in cutaneous squamous cell carcinoma
Source: J Transl Med. 2024 Apr 11;22:347. doi: 10.1186/s12967-024-05116-8 (PMC11010364; doi:10.1186/s12967-024-05116-8)
Supplement: Supplementary file 6 — Supplementary Material 6 [file 12967_2024_5116_MOESM6_ESM.docx]

Dear editors and reviewers,

We sincerely appreciate the opportunity to revise our manuscript titled "THOC7-AS1/OCT1/FSTL1 axis promotes EMT and serves as a therapeutic target in cutaneous squamous cell carcinoma" for potential publication in the Journal of Translational Medicine. Your thoughtful comments and constructive feedback have greatly contributed to enhancing the quality and impact of our research. Below, we outline the changes we made in response to your valuable suggestions.

**Comments:**

**Reviewer #1:**

The authors should justify and expand the information on the advantages of this work for biomedical applications. Authors should specify the main experimental conditions used based on the evidence from the literature. Where they briefly describe the most important data reported in the literature in a homogeneous manner and reinforce the relevance of this method as novel alternatives. Authors should discuss whether the use of work represents a solid alternative to existing therapeutics. Also, please discuss the use of method using green nanomaterials. The article lacks some background information on the significance of studying. Providing a brief introduction or literature review on the potential sources would enhance the relevance of the study. I recommend that the authors cite and comment on other previous studies that evaluated the effect of this work. There are excellent studies that should be mentioned in the introduction.

Thank you very much for your comments and suggestions. Your insights have been invaluable in refining the direction and concepts of the article, as well as incorporating crucial elements that were missing. We have implemented relevant changes and improvements in both the introduction and discussion sections of the manuscript.

**Response:**

**Q:** The authors should justify and expand the information on the advantages of this work for biomedical applications.

**A:** Thank you for your valuable suggestion. In response, we've included a dedicated paragraph in the discussion section highlighting the biomedical applications of our research. Key advantages encompass: a. Up-regulated FSTL1 expression as a potential diagnostic marker for cSCC due to its pro-cancer role. b. Contribution to the identification of novel clinical therapeutic targets, reducing cytotoxicity associated with traditional systemic drug therapy. c. Detailed elucidation of the molecular mechanism governing FSTL1 expression regulation, specifically through the THOC7-AS1/OCT1/FSTL1 axis, paving the way for the design of more targeted drugs to impede the progression of cSCC.

**Q:** Authors should specify the main experimental conditions used based on the evidence from the literature. Where they briefly describe the most important data reported in the literature in a homogeneous manner and reinforce the relevance of this method as novel alternatives.

**A:** Certainly. In response to your request, we will elaborate on our experimental conditions in the discussion section. Our study employed conventional experimental and analytical methods, grounded in evidence from the literature. The identification of key molecular FSTL1 in cSCC was based on comprehensive data from public databases. To validate its aberrant upregulation in cSCC, we utilized a combination of qPCR, western blot, IHC, and ICC techniques across both cell lines and tissue samples. Furthermore, our analysis of clinical samples underscored the correlation between elevated FSTL1 expression and adverse parameters such as tumor size and local infiltration in cSCC patients, affirming its pro-oncogenic role. Functional experiments, encompassing cell transfection, clone formation, CCK8 assays, scratch assays, transwell assays, flow cytometry for apoptosis and cell cycle, and xenograft tumor formation, were conducted to provide comprehensive evidence of FSTL1's involvement in promoting cell proliferation, migration, and invasion in cSCC.

Molecular insights were further elucidated through a battery of techniques, including GSEA enrichment analysis, qPCR, western blot, co-IP, ICC, RNAseq, FISH, RIP, RNA pull down, ChIP, dual-luciferase reporter gene assay, ASO, and cell function recovery experiments. These methodological choices were made to ensure the reproducibility and reliability of our research, reinforcing the relevance of our approach as a novel alternative in the field.

**Q:** Authors should discuss whether the use of work represents a solid alternative to existing therapeutics.

**A:** Yes. Our study contributes novel insights that hold promise as a solid alternative for the treatment of squamous carcinoma. By identifying the pro-oncogenic role of FSTL1 in cSCC and elucidating the THOC7-AS1/OCT1/FSTL1 mechanism governing EMT and tumor progression, our research represents a significant advancement. This innovative approach opens avenues for targeted therapies against FSTL1 or the THOC7-AS1/OCT1/FSTL1 axis, offering potential benefits for late-stage cSCC patients. These targeted treatments, whether administered independently or in combination with existing cytotoxic drug therapies, have the potential to enhance treatment efficacy, alleviate drug side effects, and expand therapeutic options. Nonetheless, further assessment is essential to gauge their effectiveness and potential side effects. Notably, the pharmaceutical company's registration of the FSTL1-targeting drug PFI-103, with ongoing exploration in osteosarcoma and solid tumors, serves as a robust reference, underlining the relevance and potential applicability of our findings in broader therapeutic contexts.

**Q:** Also, please discuss the use of method using green nanomaterials. The article lacks some background information on the significance of studying. Providing a brief introduction or literature review on the potential sources would enhance the relevance of the study. I recommend that the authors cite and comment on other previous studies that evaluated the effect of this work. There are excellent studies that should be mentioned in the introduction.

**A:** Certainly. Your suggestion has been incorporated. In the discussion section, we have addressed the significance of the method using green nanomaterials by citing relevant studies recommended by the reviewers. Additionally, we have provided a brief background on green silver nanoparticles within the existing literature, establishing the context for the study. The discussion highlights the potential connections of green silver nanoparticles to our research and explores potential biological applications. To underscore the significance of utilizing green nanosilver particles for targeted therapy, we draw attention to research indicating that AgNPs synthesized through biological resources exhibit biocompatibility and possess robust antimicrobial, antioxidant, anti-inflammatory, and anti-tumor properties. These nanoparticles find applications in diagnostics, cell labeling, biomarkers, drug delivery, cancer treatment, and water purification. Noteworthy studies have demonstrated the anti-cancer effects of AgNPs obtained through green synthesis on various cancer cell lines, achieving inhibitory rates ranging from 25% to 81% at a concentration of 25 μg/mL. Building on these properties, our study proposes the labeling or delivery of the FSTL1-targeting drug or ASO for the THOC7-AS1/OCT1/FSTL1 axis using nanosilver particles to potentially enhance their anti-cancer effects. This addition strengthens the relevance of our study within the broader context of green nanomaterials and their applications in biomedicine.

**Reviewer #2:**

-please overexpress the FSTL1 in human cSCC cell lines and then investigate the effect of FSTL1 in tumor inhibition of squamous cell carcinoma.
- please perform ANNEXIN V and cell cycle
-The transwell assay method to check invasion should be modified
-Using the Immunocytochemistry (ICC) phrase instead of Immunofluorescence (IF) would be better.
- In the method section, explain that Immunohistochemistry performed for which samples.
-At the end of the introduction, please state the existing problems and the purpose of this study to solve the existing problems.

Thank you sincerely for your valuable guidance. The inclusion of pertinent experimental content has enriched our article with additional data and supporting evidence, thereby enhancing the overall persuasiveness of the ideas presented. Following your advice, we have effectively conducted the necessary supplementary experiments and acquired corresponding results.

**Q:** please overexpress the FSTL1 in human cSCC cell lines and then investigate the effect of FSTL1 in tumor inhibition of squamous cell carcinoma.

**A:** That's an insightful suggestion. We conducted FSTL1 overexpression experiments in A431 and Colo16 cells, confirming the efficacy through western blot analysis (Fig 3C-D). The FSTL1 overexpression group exhibited a significant increase in FSTL1 expression compared to the control group. Subsequently, following the successful overexpression of FSTL1 in tumor cells, we delved into cell function experiments. In clone formation assays (Fig 3G-H), the overexpression of FSTL1 resulted in a noteworthy increase in the number of cell clones compared to the control group. CCK8 experiments (Fig 3J) revealed that FSTL1 overexpression led to an augmentation in the 450 nm absorption peak, indicative of enhanced cell viability. In transwell assays and scratch assays, the overexpression of FSTL1 demonstrated a substantial increase in the number of cells traversing the matrix gel (Fig 3 M-N), accompanied by a greater migration distance compared to the control group (Fig 3 Q-R). Collectively, these experiments indicate that in cutaneous squamous cell carcinoma, FSTL1 overexpression amplifies the proliferation, migration, and invasion capabilities of tumor cells, thereby promoting tumor progression.

**Q:** please perform ANNEXIN V and cell cycle
**A:** OK. We carried out assessments to examine the impact of FSTL1 on apoptosis and the cell cycle. Our results indicate that the knockdown of FSTL1 did not induce notable changes in apoptosis when compared to the control group (Fig S1 A-B). Additionally, there were no significant alterations observed in the cell cycle following the knockdown of FSTL1 (Fig S1 C-D). These findings suggest that, under the conditions examined in this study, FSTL1 may not play a prominent role in regulating apoptosis or influencing the progression of the cell cycle.

**Q:** The transwell assay method to check invasion should be modified

**A:** Absolutely, the description of the experimental steps in the transwell assay required modification. We have revised the procedural details for this experiment, and the updated steps are as follows:

"For the investigation of cellular invasion, the upper chambers were pre-coated with matrix gel (354248; Corning, Inc.) following the provided instructions prior to cell seeding. Transwell chambers (pore size, 8 µm; Corning, Inc.) were assembled with 500μL of serum-free DMEM in the upper chamber and 500μL of 20% FBS DMEM in the lower chamber. cSCC cells were then seeded in the upper chamber. After 24 hours, the cells were fixed using 4% paraformaldehyde, stained with crystal violet, and imaged using a Nikon microscope. Subsequently, cell counting was performed."

**Q:** Using the Immunocytochemistry (ICC) phrase instead of Immunofluorescence (IF) would be better.

**A:** Thank you for your suggestion. We will make the necessary adjustments in the manuscript, replacing "Immunofluorescence (IF)" with "Immunocytochemistry (ICC)."

**Q:** In the method section, explain that Immunohistochemistry performed for which samples.
**A:** In the methodology section, it is important to note that Immunohistochemistry was conducted to assess the expression levels of specific markers. This analysis was performed on human cutaneous squamous cell carcinoma (cSCC) tissue samples for FSTL1 and ZEB1. Additionally, the evaluation extended to nude mouse subcutaneous tumor tissue samples, encompassing markers such as FSTL1, Ki67, ZEB1, E-cadherin, N-cadherin, and Vimentin.

**Q:** At the end of the introduction, please state the existing problems and the purpose of this study to solve the existing problems

**A:** That's a valuable suggestion. In order to provide context for the purpose of our study, we identified existing problems in the field: a. Advanced cutaneous squamous cell carcinoma is characterized by high invasiveness, easy metastasis, and limited therapeutic options. b. Conventional therapies are associated with significant side effects, and the need for new drug development is urgent. c. The molecular regulatory mechanism of FSTL1 is unknown, making it a potential target for evaluation. To address these challenges, we have incorporated the following at the end of the introduction: "Late-stage cutaneous squamous cell carcinoma is characterized by strong invasiveness, rapid progression, and a high risk of recurrence and metastasis. Surgical intervention is often not feasible at this stage, and existing systemic treatment options, primarily cytotoxic chemotherapy and EGFR inhibitor therapy, are relatively limited. While PD-1 immune checkpoint inhibitors show promise in clinical and preclinical trials, novel therapeutic strategies are scarce. The paucity of targeted treatment options for cSCC underscores the pressing need for exploring new therapeutic targets. This study aims to address these challenges from a clinical perspective. FSTL1, identified as a key signaling molecule in our cSCC research, has demonstrated a crucial role in the progression of gastric and hepatic cancers. However, its regulatory mechanisms in cutaneous squamous cell carcinoma remain unclear. Therefore, our experiments will delve into the expression and regulatory mechanisms of FSTL1, elucidating its pro-carcinogenic effects on cSCC.

Additionally, ongoing preclinical trials exploring the FSTL1-targeting drug PFI-103 for osteosarcoma and solid tumors provide a basis for our research. We anticipate that our experimental findings will further substantiate the clinical therapeutic value of FSTL1, offering potential breakthroughs in cancer treatment."

Thank you for your thoughtful comments and valuable suggestions. If you have any further questions or require additional information, please do not hesitate to reach out. We sincerely appreciate your careful consideration of our paper and eagerly anticipate hearing from you soon.

Thank you once again.

Best wishes,

Pihong Zhang

Xiangya Hospital, Central South University.

Xiangya Road No.88, Changsha, Hunan province, China.

Email: zphong2022@126.com
